# Aluminium-induced component engineering of mesoporous composite materials for low-temperature NH_3_-SCR

**DOI:** 10.1038/s42004-020-0311-4

**Published:** 2020-05-27

**Authors:** Ge Li, Baodong Wang, Ziran Ma, Hongyan Wang, Jing Ma, Chunlin Zhao, Jiali Zhou, Dehai Lin, Faquan He, Zhihua Han, Qi Sun, Yun Wang

**Affiliations:** 1 National Institute of Clean-and-Low-Carbon Energy, Beijing, 102211 China; 2 Institute for Sustainable Energy and Resources, Qingdao University, Shandong, 266071 China; 3 Centre for Clean Environment and Energy, Gold Coast Campus, Griffith University, Southport, QLD 4222 Australia

**Keywords:** Heterogeneous catalysis, Catalyst synthesis

## Abstract

Supported Mn_2_O_3_ is useful in achieving high dinitrogen selectivity at low temperature during ammonia-selective catalytic reduction (SCR). However, its controlled synthesis is challenging when the supporting material is the conventional pure silicon SBA-15 mesoporous molecular sieve. Here we show that silicon and aluminium in fly ash, the solid waste produced by coal-fired power plants, can be used to synthesize an Al-SBA-15 mesoporous molecular sieve support, which can guide the growth of Mn_2_O_3_ in the as-synthesized Fe-Mn/Al-SBA-15 NH_3_-SCR catalyst. Its superior catalytic performance is demonstrated by the high NO_x_ conversion (≥90%) and selectivity (≥86%) at low temperatures (150–300 °C). The combined theoretical and experimental results reveal that the introduction of Al induces the growth of Mn_2_O_3_ catalysts. Our findings, therefore, provide a strategy for the rational design of low-temperature NH_3_-SCR catalysts through dopant-induced component engineering of composite materials.

## Abstract (web summary)

Low-temperature manganese-based denitrification catalysts require a balance between high reduction reactivity and selectivity. Here aluminium in a mesoporous molecular sieve induces the growth of manganese oxide to obtain an iron-manganese catalyst which achieves high efficiency at temperatures of 150–300 °C.

## Introduction

The large amount of nitrogen oxides (NO_x_) produced by the combustion of fossil fuels (mainly coal, oil, and natural gas) is an important precursor of fine particles and an important cause of smog^1^. China’s smog pollution has had a serious impact on the global environment^2,3^. Ammonia-selective catalytic reduction (NH_3_-SCR) of NO_x_ is technically an efficient way to control and reduce stationary NO_x_ emissions, such as from power plants, industrial boilers, and kilns. However, the conventional commercial V_2_O_5_-WO_3_/TiO_2_ catalyst is incapable of reducing NO_x_ by NH_3_ when the flue gas temperature is ≤300 °C^4–6^. Moreover, the key active ingredient V, a toxic and harmful element to the environment and human health, might be leached out to further pollute the environment. It is imperative to develop an environmentally friendly catalyst to efficiently reduce NO_x_ at low temperatures.

In recent years, low-cost and nontoxic zeolite catalysts have become a favorable SCR catalyst carrier^7–12^. Among all the studied zeolites, SBA-15 mesoporous molecular sieves (MMS) have attracted the most attention due to their larger specific surface area (690–1040 m^2^/g), larger pore size (4.6–30 nm), and better hydrothermal stability^13–18^. In our previous work^19–21^, Fe–Mn/SBA-15 catalysts were prepared using a fly ash-derived SBA-15 MMS as a support and tested for NH_3_-SCR. The 11.2Fe-11Mn/SBA-15 catalyst exhibits high NH_3_-SCR activities at 150–250 °C. However, this catalyst favors N_2_O formation. Previous studies reveal that the polymorph of MnO_x_ including MnO_2_, Mn_5_O_8_, Mn_2_O_3_, Mn_3_O_4_, and MnO is critical to the selectivity of the catalyst^22^. Among them, Mn_2_O_3_ has the best selectivity for the nontoxic N_2_ production^22^. However, it is difficult to synthesize Mn_2_O_3_ on the SBA-15 MMS using impregnation method. Therefore, innovative pathways are needed.

From the molecular design point of view for the NH_3_-SCR catalyst, the denitrification catalyst needs to have suitable acid-base and redox properties^23,24^. There are a large number of hydroxyl structures on the surface of SBA-15 MMS. Doping with Al can increase the acidity of the molecular sieves. The Brønsted (B) acid sites on the framework are generated, and a Lewis (L) acid is produced by extra-framework Al (EFAL) outside the pores. On the other hand, the surface can be loaded with active components to build the catalyst’s redox properties. In our present work, we theoretically predicted that the introduction of Al could induce the formation of Mn_2_O_3_, as elucidated in detail later. However, efficiently doping Al into the framework of SBA-15 zeolites is difficult. In recent years, both one-step copolycondensation and post-synthesis methods have been employed to prepare Al-SBA-15 MMS^25–30^. In the one-step copolycondensation method, Al exists in the form of a complex, which is difficult to condense with the positively charged Si in the strong acid system. Therefore, it is challenging to successfully insert Al cations into the silica skeleton in this method. While more Al cations can be introduced in the post-synthesis method by strictly controlling the pH of the system and reducing the hydrolysis of the Al salt, a large amount of EFAL is swept away during the washing process, which reduces the total acidity of the Al-SBA-15 catalyst. Therefore, the development of an efficient way for effectively doping Al in SBA-15 with suitable acidity holds the key to the molecular design of high-performance NH_3_-SCR catalysts.

In this study, fly ash, solid waste produced by coal-fired power plant, is used as the raw material for the preparation of SBA-15 MMS and AlCl_3_·6H_2_O powders. An impregnation method is subsequently used to synthesize Al-SBA-15 MMS by controlling the pH value. After that, an impregnation method is used to prepare the Fe–Mn/Al-SBA-15 catalyst, which exhibits a high overall performance for NH_3_-SCR denitrification at low temperature. In addition, the denitrification reaction mechanism for the Fe–Mn/Al-SBA-15 catalyst at 200 °C is investigated by in situ infrared (IR) spectroscopy. Our combined experimental characterization and computational results reveal that the superior performance of the Fe–Mn/Al-SBA-15 catalyst can be ascribed to the controllable growth of Mn_2_O_3_ polymorph due to the Al-induction.

## Results

### Characterizations of fly ash-derived Al-SBA-15 MMS

**Fig. 1:**
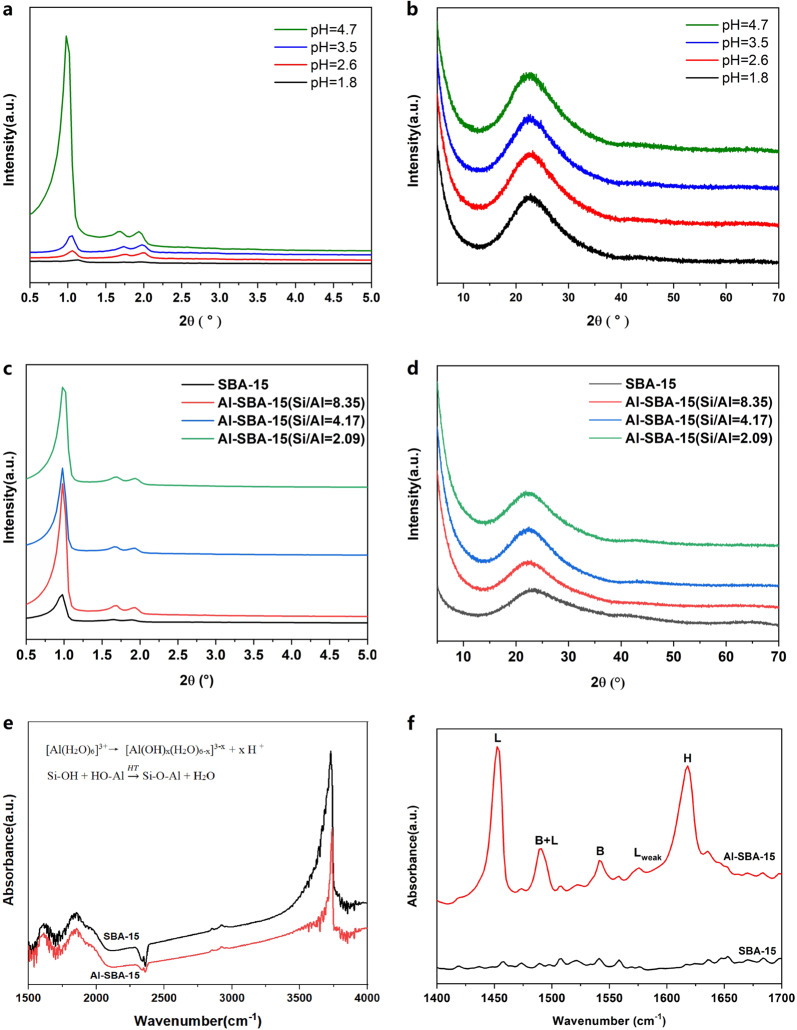
Structural characterization and IR spectra of Al-SBA-15 molecular sieves. **a** SXRD patterns of Al-SBA-15 MMS prepared under various pH values. **b** XRD patterns of Al-SBA-15 MMS prepared under various pH values. **c** SXRD patterns of Al-SBA-15 MMS prepared under various Si/Al ratios. **d** XRD patterns of Al-SBA-15 MMS prepared under various Si/Al ratios. **e** In situ FTIR with transmission mode of SBA-15 and Al-SBA-15. **f** IR spectra of pyridine adsorbed on SBA-15 and Al-SBA-15.

Since the pH value is crucial for the aluminum salt hydrolysis and Si–O–Al bonds formation, we first investigated the effect of the pH value on the synthesis of fly ash-derived Al-SBA-15 MMS. 1 g of the fly ash-derived Si-SBA-15 MMS and 1 g of fly ash-derived AlCl_3_·6H_2_O powder were added to 50 mL of absolute ethanol (pH = 4.7), 50 mL of H_2_O (pH = 3.5), 100 mL of H_2_O (pH = 2.6), and 150 mL of H_2_O (pH = 1.8), respectively. The impregnation experiment was carried out by magnetic stirring at 80 °C for 12 h. After spin-drying and calcination at 550 °C for 5 h at a heating rate of 5 °C/min, Al-SBA-15 MMS were synthesized. Figure 1a, b shows the small angle X-ray diffraction (SXRD) and X-ray diffraction (XRD) patterns of the Al-SBA-15 MMS prepared at different pH values. When the pH value was >3.5, the SXRD pattern of the obtained Al-SBA-15 MMS possesses a prominent peak at 2θ = 0.8° and two small peaks at 2θ = 1.6° and 1.8°, which have been indexed to the (100), (110), and (200) diffraction peaks of the SBA-15 XRD pattern, respectively^13^. This suggests that the original pore characteristic peak of the SBA-15 MMS maintains in this pH range. However, when the pH values were 2.6 and 1.8, the diffraction peak of the molecular sieve (100) crystal plane was obviously weakened, which indicated that the ordering of the molecular sieve pores was significantly reduced. The isoelectric point of SiO_2_ is 1.7–3.5^28,29^. It suggests that the surface of SiO_2_ is negatively charged when the pH value of the solution is higher than 3.5. Consequently, Al^3+^ can be adsorbed through electrostatic interaction, which is beneficial for the formation of Si–O–Al bonds. The weakly acidic environment produced by the hydrolysis of inorganic aluminum salts (the pH value is close to the isoelectric point of silicon oxide) can also reduce the polymerization rate of silanol groups in the pore walls of SBA-15 material, which leads to a larger amount of silanol groups on the pore walls of the material. The silanol groups can interact with Al–OH in solution to form a Si–O–Al bond through high-temperature polymerization. Therefore, uniformly grafting Al to the silicon oxide mesoporous skeleton of the material is achieved when the pH value is higher than the isoelectric point of silicon oxide.

Figure 1c, d shows the small-angle powder XRD patterns and wide-angle XRD patterns of Si-SBA-15 before and after post-synthesis alumination under various Si/Al ratios. The XRD peaks were indexed to a hexagonal lattice with a *d*_100_ spacing of 9.7 nm, which corresponded to a unit cell parameter *a*_0_ of 11.2 nm by using the formula $a_0 = \frac{{2d_{100}}}{{\sqrt 3 }}$ ^27^. The well-defined XRD patterns showed that all the samples retained the characteristic hexagonal mesostructure of SBA-15 after alumination. No Al_2_O_3_ diffraction peaks were observed in the wide-angle XRD patterns regardless of the amount of incorporated Al. This indicated that all the Al were incorporated into the SBA-15 skeleton or highly dispersed as non-skeleton Al. It, therefore, proves that Al-SBA-15 MMS have been successfully synthesized.

To avoid the influence of moisture on the surface hydroxyl test of the molecular sieves, we used in situ IR spectroscopy in the transmission mode to characterize the surface hydroxyl groups of SBA-15 and Al-SBA-15 MMS. Figure 1e shows that, compared with the SBA-15 MMS, the isolated silanol stretching vibration peak of the Al-SBA-15 (Si/Al = 4.17) MMS is obviously changed near 3740 cm^−1^ with considerably small the peak intensity and peak area. This phenomenon suggested that Al doping modifies the surface silanol groups of SBA-15, as reported in the literature^27–29^.

The FTIR spectra for pyridine (Py) adsorbed on SBA-15 and Al-SBA-15 (Si/Al = 4.17) are shown in Fig. 1f. The results reveal that pure Si SBA-15 is not acidic. After Al doping, Al-SBA-15 exhibits two strong absorption peaks at 1452 and 1618 cm^−1^. The absorption peak at 1452 cm^−1^ arose from the complex (LPy) produced by interactions of Py molecules with Lewis acids, and the peak at 1618 cm^−1^ resulted from pyridine (HPy) adsorbed on surface hydroxyl groups through hydrogen bonds. The absorption peak at 1541 cm^−1^ corresponds to Brønsted acidic centers. The peak at 1490 cm^−1^ corresponded to a combination of Lewis and Brønsted acids. The formation of Brønsted acidic centers was attributed to Si–OH–Al formed by polymerization of Al–OH produced by hydrolysis of the Al-containing solution (AlCl_3_) and Si–OH in the pore walls of SBA-15 in the synthesis system. The Py-IR results demonstrate that the incorporation of Al can significantly enhance the surface concentrations of L and B acids to provide favorable conditions for its applications as a catalyst carrier.

**Fig. 2:**
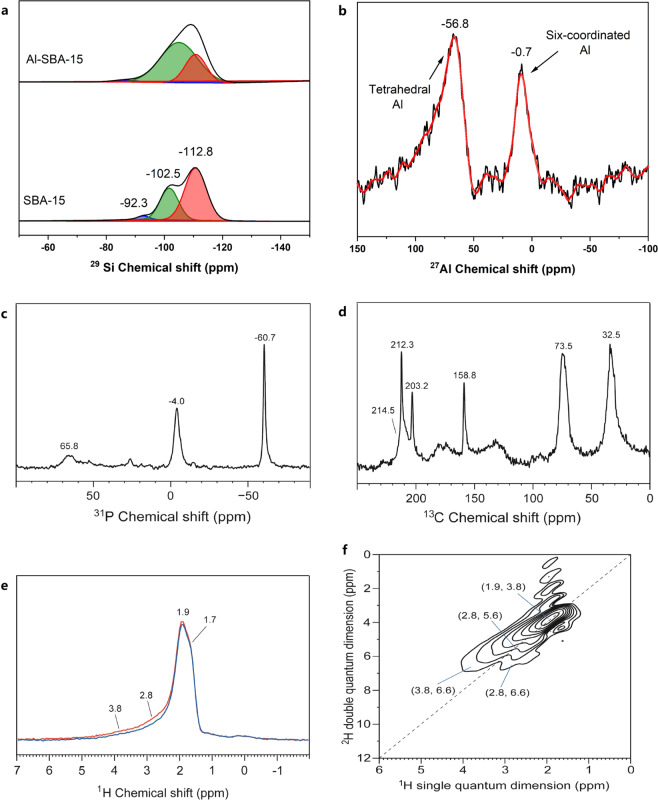
NMR spectra of Al-SBA-15 samples. **a** ^29^Si MAS spectra (compared with SBA-15). **b** ^27^Al MAS spectra. **c** ^31^P MAS NMR spectra. **d** ^13^C CP/MAS NMR spectra. **e** ^1^H/^27^Al TRAPDOR NMR spectra. **f** ^1^H DQ-MAS NMR spectra.

^29^Si and ^27^Al magic angle spinning (MAS) nuclear magnetic resonance (NMR) spectroscopies were used to investigate the Al-SBA-15 samples to confirm the effects of Al doping on silanol of Si species in the samples. Figure 2a shows that the spectrum of Al-SBA-15 is significantly broadened, compared with the spectrum of SBA-15, indicating that Si-Al dipole action occurs^28^. The ^29^Si MAS NMR spectrum of SBA-15 showed three chemical shifts at *δ* = − 92.3, −102.5, and −112.8, which corresponds to Si(OSi)_2_(OH)_2_ (Q^2^, chain structure), Si(OSi)_3_(OH) (Q^3^, rack structure), and Si(OSi)_4_ (Q^4^, island structure), respectively^29^. Q^3^ is mainly on the surface of the tunnel and is important for material modification. The fractionated peak fitting results suggest that the proportion of Q^3^ is significantly increased after Al doping, indicating that the surface Si–OH is significantly increased. When Al^3+^ substitutes Si^4+^ without changing the oxygen coordination number, a hydroxyl group on Al^3+^ is required to maintain the charge neutral condition. Consequently, a Brønsted acidic center is generated. This is the reason for the increase in Si–OH after Al doping. The framework Si:Al ratio in the MMS can be calculated from Q^3^ and Q^4^. The framework Si:Al ratio for the Al-SBA-15 MMS sample was calculated to be 14.64 by using the equation^28,29^:

1 $$\frac{{\rm{Si}}}{{\rm{Al}}} = \frac{{\mathop {\sum }\nolimits_{i = 0}^4 {\rm{I}}_{\rm{Si}[{\it{n}}Al]}}}{{\mathop {\sum }\nolimits_{i = 0}^4 0.25n{\rm{I}}_{\rm{Si}[{\it{n}}Al]}}}.$$

The coordination environments of Al in the Al-SBA-15 materials were determined by ^27^Al MAS NMR spectroscopy. The spectra of the Al-SBA-15 materials show two resonances (Fig. 2b). The resonance at 0 ppm, which arises from six-coordinated Al, is more intense than the resonance at 56 ppm, which can be assigned to the tetrahedral framework Al formed in the mesoporous walls of the material^31^. This demonstrates that a post-synthesis procedure can be used to graft Al–OH species into the mesoporous walls of SBA-15. And the formation of Si–O–Al bonds increases the relative amount of tetrahedral Al in the pore walls of the resulting material. These results combined with the data obtained by Py-IR spectroscopy suggest that Al atoms were successfully incorporated into the porous surface of the mesoporous SBA-15 structure by a post-alumination procedure.

The relative value of the ^31^P chemical shift can be used to determine the acidic strength of a catalytic material. It is generally believed that the trimethylphosphine molecule (TMP) interacts with a Brønsted acid to form TMPH^+^ ions, whose chemical shift is ~0 ppm in the single-pulse ^31^P spectrum. Although the chemical shift of the TMP molecule adsorbed on a Lewis acid is between −32 and −58 ppm, the chemical shift of physically adsorbed TMP is approximately −60 ppm^32^. Figure 2c shows the single pulsed ^31^P spectrum of TMP adsorbed on Al-SBA-15. Two signals were well resolved at −4.0 and −60.7 ppm. When the TMP was removed under vacuum at 80 °C for 2 h, the peaks at −4 and −60 ppm were still present, which indicates the presence of a stable Lewis acid in the Al-SBA-15 sample. This is consistent with the Py-IR results. The signal at 65.8 ppm showed that TMP was oxidized to TMPO adsorbed on the Brønsted acid of the Al-SBA-15 sample. Meanwhile, it was also indicated that the Al-SBA-15 sample prepared by the impregnation method had both Lewis acid and Brønsted acid. According to the integral area of the two peaks, the L acid had a stronger acidic strength than the Brønsted acid. The Al-SBA-15 sample prepared using the ion exchange method in previous studies^28,29^ only had Brønsted acid caused by the skeleton Al. While the Al-SBA-15 sample prepared by the impregnation method contained non-framework Al.

Previous studies have confirmed that the isotropic chemical shift of the 2-^13^C-acetone carbonyl carbon can be used to measure the strength of various solid acids^33,34^. After the molecular sieve adsorbs acetone, a stronger Brønsted acidity will result in a greater chemical shift to the lower field. Therefore, acetone is a useful probe molecule to measure the relative acidic strength of a solid acid. The ^1^H/^13^C CP MAS spectrum after adsorption of acetone by the Al-SBA-15 MMS is shown in Fig. 2d. The three peaks at 212.3, 73.5, and 32.5 ppm correspond to the products of dimerization or trimerization of acetone molecules, in addition to the peak at 214 ppm, which was considered as the signal of unreacted acetone adsorption at unequal acid sites^31^. Because the Al-SBA-15 MMS showed no peak at 232 ppm, this indicated that the Al-SBA-15 MMS had no more acidic acid sites. The Brønsted acidity of the signal at 218 ppm was also weaker than that of HZSM-5 (chemical shift was 223 ppm)^31^. According to the integrated area of the peak, the Lewis acid strength of the Al-SBA-15 MMS was greater than the acidic strength of the Brønsted acid, which is consistent with the results of TMP-^31^P NMR and Py-IR.

To establish a correlation between the various hydroxyl groups and Al, we performed ^1^H/^27^Al transfer of population in double resonance (TRAPDOR) experiments on the Al-SBA-15 samples. Figure 2e shows a weak signal at 2.8 ppm and a strong signal at 1.9 ppm, which were ascribed to AlOH groups and SiOH in the vicinity of Al atoms, respectively. This structure was similar to that of germinal or hydrogen-bonded hydroxyl groups^33^. The peak at 3.8 ppm was assigned to the protons at the Brønsted acid sites^33^, which indicated that Al was tetrahedrally coordinated in the silica framework. Moreover, the concentration of the bridged hydroxyl groups was low with respect to Si–OH.

It is widely believed that the non-skeleton is mainly composed of aluminum oxide ions (such as [AlO]^+^, [Al(OH)_2_]^+^, [AlOH]^2+^) and some electrically neutral species (such as AlOOH, Al(OH)_3_, and polymeric Al_2_O_3_)^33^. Whether there is a synergistic effect between the Brønsted acid associated with the framework aluminum and the Lewis acid outside the framework can be verified by two-dimensional ^1^H double quantum (DQ)-MAS NMR experiments. Figure 2f shows a two-dimensional ^1^H DQ-MAS NMR image of an Al-SBA-15 molecular sieve. Cross-peak pairs at (3.8, 6.6) ppm and (2.8, 6.6) ppm revealed the spatial proximity between the non-framework aluminum hydroxy (Lewis acid) and the framework Brønsted acid proton in the Al-SBA-15 MMS. At the same time, two sets of autocorrelation peaks (2.8, 5.6) ppm and (1.9, 3.8) ppm were observed, which indicated that the same hydroxyl groups were not isolated from each other but were spatially adjacent (distance less than 5 Å). The autocorrelation peak at (2.8, 5.6) ppm arose from extra-framework AlOOH or [Al(OH)]^2+^ in close proximity or EFAL species containing more than one hydroxyl group, such as Al(OH)_3_ and [Al(OH)_2_]^+^. The spatial proximity of the Lewis acid and the Brønsted acid increased the overall acidity of the catalyst, which is favorable for the SCR reaction.

Supplementary Fig. 1 shows that the N_2_ adsorption–desorption isotherms of all the SBA-15 MMS, whether pure silica or Al-doped molecular sieves, were typical type IV isotherms with H1-type hysteresis loops. The pore volume, pore size, and Brunauer–Emmett–Teller (BET) specific surface area decreased with increasing amount of grafted Al. The details of the N_2_ adsorption–desorption isotherms of all samples are shown in Supplementary Fig. 1, Supplementary Table 1, and Supplementary Note 1 of the Supplementary Information.

**Fig. 3:**
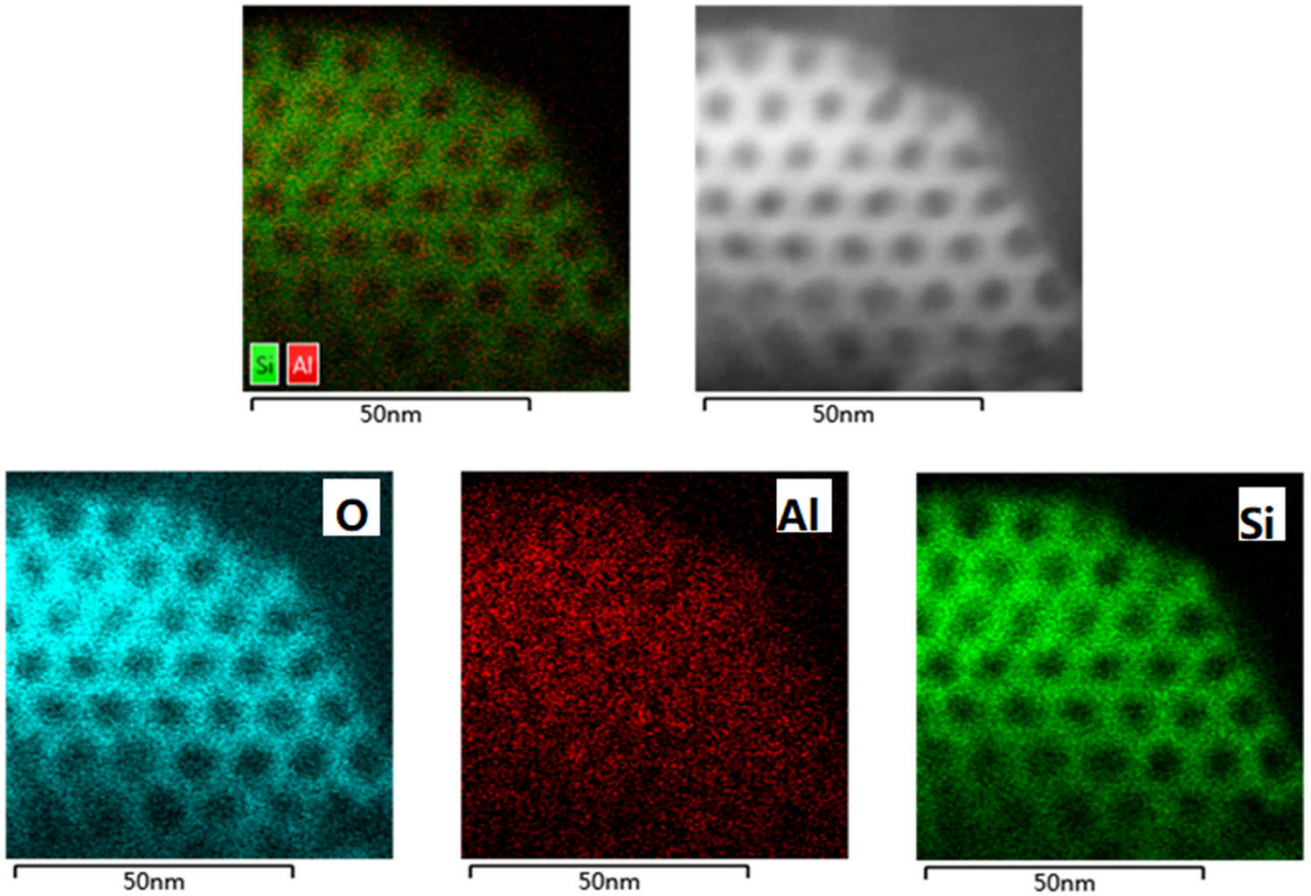
HAADF-STEM images of the Al-SBA-15 MMS. **a** the Al-SBA-15 sample in the [100] orientation. **b** the image of the same area, showing the contrasts of the different types of metal species. **c** O element distribution images of the same area, which shows the presence of O in the framework. **d** Al element distribution images of the same area, which shows Al species are uniformly distributed on the molecular sieve, both on the framework and outside the framework. **e** Si element distribution images of the same area, which shows the presence of Si in the framework.

Figure 3 is the high-angle annular dark field scanning transmission electron microscopy (HAADF-STEM) images of Al-SBA-15 MMS, which evidences that the resulting Al-SBA-15 MMS had a perfect hexagonal porous structure (Fig. 3a). And Al species are uniformly distributed on the molecular sieve, both on the framework and outside the framework (Fig. 3d). No large Al species was observed, which indicates EFAL evenly distributed.

The hydrothermal stability of obtained Al-SBA-15 MMS was examined at 100 and 600 °C according to previous studies^13–15^. The structure and specific surface area of Al-SBA-15 samples after hydrothermal aging were characterized by XRD and N_2_ adsorption–desorption (see Supplementary Figs. 2 and 3, Supplementary Table 2, and Supplementary Note 2), respectively. The results show that all the Al-SBA-15 MMS basically maintained their original morphology after hydrothermal treatment. However, the micropore structure of the Al-SBA-15 molecular sieve disappeared and the mesopore structure was partially destroyed after hydrothermal treatment at 600 °C. Our findings are consistent with previous studies^13–15^.

### Properties of Fe–Mn/Al-SBA-15 catalysts

**Fig. 4:**
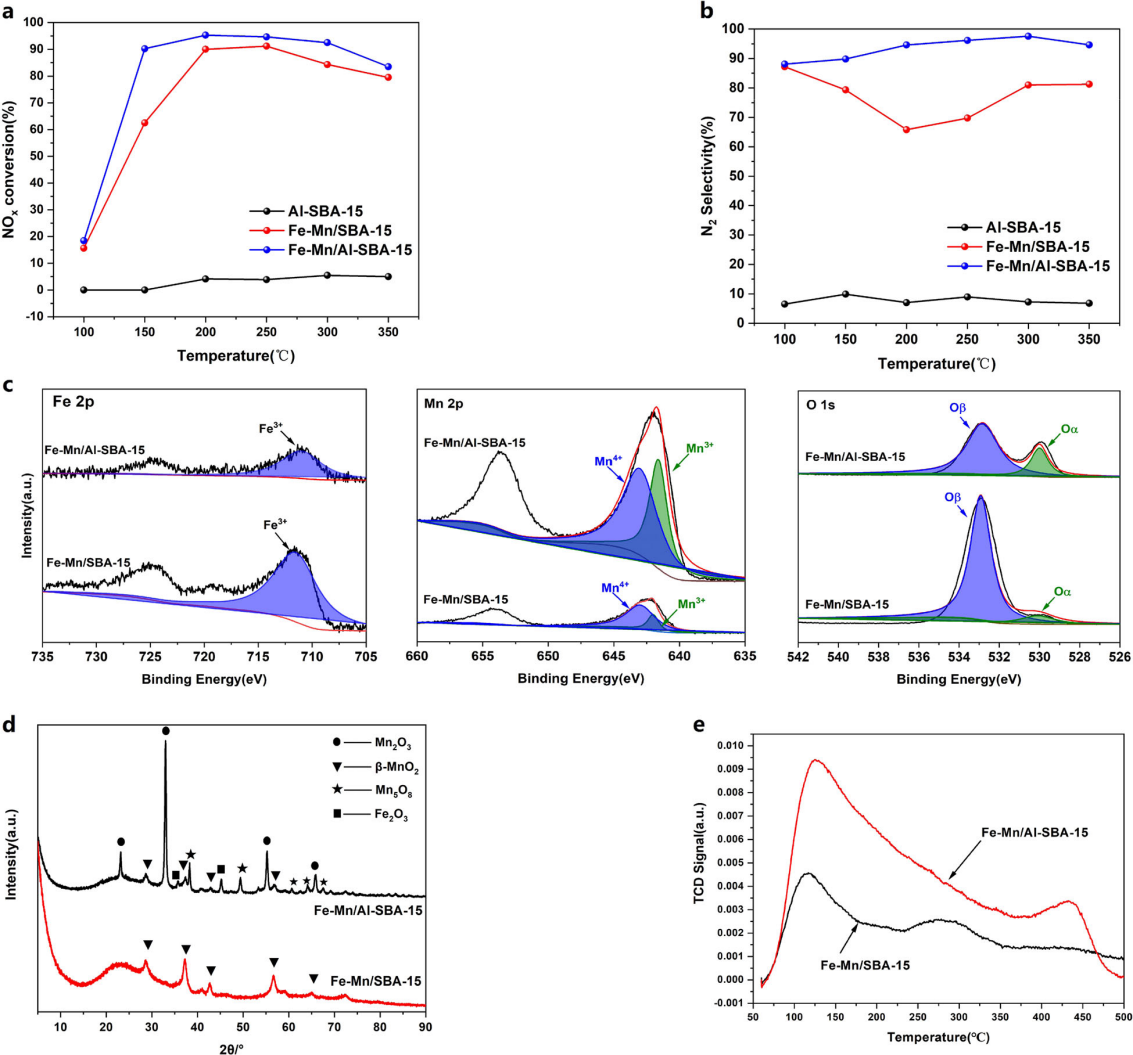
NH_3_-SCR performances and characterization of Fe–Mn/Al-SBA-15 and Fe–Mn/SBA-15 catalysts. **a** NO_x_ conversion. **b** N_2_ selectivity. **c** XPS spectra. **d** XRD patterns. **e** NH_3_-TPD curves.

The NO_x_ conversion of the Fe–Mn/Al-SBA-15 catalyst was significantly improved at low temperature (Fig. 4a). At 150 °C, the NO_x_ conversion was increased from 62.8 to 91.1%, and the efficient denitrification temperature window was widened. Figure 4b shows that the N_2_ selectivity of the catalyst was significantly improved after doping with Al, and the N_2_ selectivity was higher than 85% at 100–350 °C. From the XPS results shown in Fig. 4c, after Al doping, the valence state of Fe was not changed. However, the valence state of Mn was greatly changed with more Mn^3+^. In combination with Table 1, the proportion of chemisorbed oxygen (O_*β*_) and lattice oxygen (O_*α*_) were also reduced. The XRD patterns shown in Fig. 4d demonstrate that β-MnO_2_ is the only Mn species on the surface of Fe–Mn/SBA-15. In addition, there is no diffraction peak of Fe_2_O_3_, which indicates that Fe_2_O_3_ was highly dispersed on the surface of the molecular sieve. In the Fe–Mn/Al-SBA-15 catalyst, a large amount of Mn_2_O_3_ was formed, which indicated that the Al doping can induce the transformation of Mn oxide polymorphs. Figure 4e shows the NH_3_-temperature programmed desorption (TPD) curve for the Fe–Mn/Al-SBA-15 catalyst and Fe–Mn/SBA-15 catalyst. The desorption peaks at 100–250, 280–330, and 380–500 °C were attributed to desorption of physisorbed NH_3_, NH_3_ bound to weak Brønsted acid sites, and NH_3_ bound to strong Brønsted and Lewis acid sites, respectively, on the catalyst surface^6^. The total areas of peaks for Fe–Mn/Al-SBA-15 were clearly bigger than that for Fe–Mn/SBA-15. This indicated that NH_3_ desorption from the Fe–Mn/Al-SBA-15 surface was easier.

**Table 1:** Binding energies (BEs) and surface atomic concentrations of Mn, Fe, and O in Fe–Mn/Al-SBA-15 catalysts.

Samples	Surface concentration (%)			Bing energy (eV)					Mn^4+^/Mn^3+^	O_*β*_/O_*α*_
	Mn2p	Fe2p	O1s	Fe 2p3/2	O1s		Mn2p3/2			
					O_*β*_	O_*α*_	Mn^4+^	Mn^3+^		
Fe–Mn/SBA-15	2.42	1.38	96.20	711.41	532.92	530.09	643.26	642.07	1.88	65.10
Fe–Mn/Al-SBA-15	16.4	0.7	82.9	711.06	532.83	530.00	643.04	642.00	1.24	2.34

**Fig. 5:**
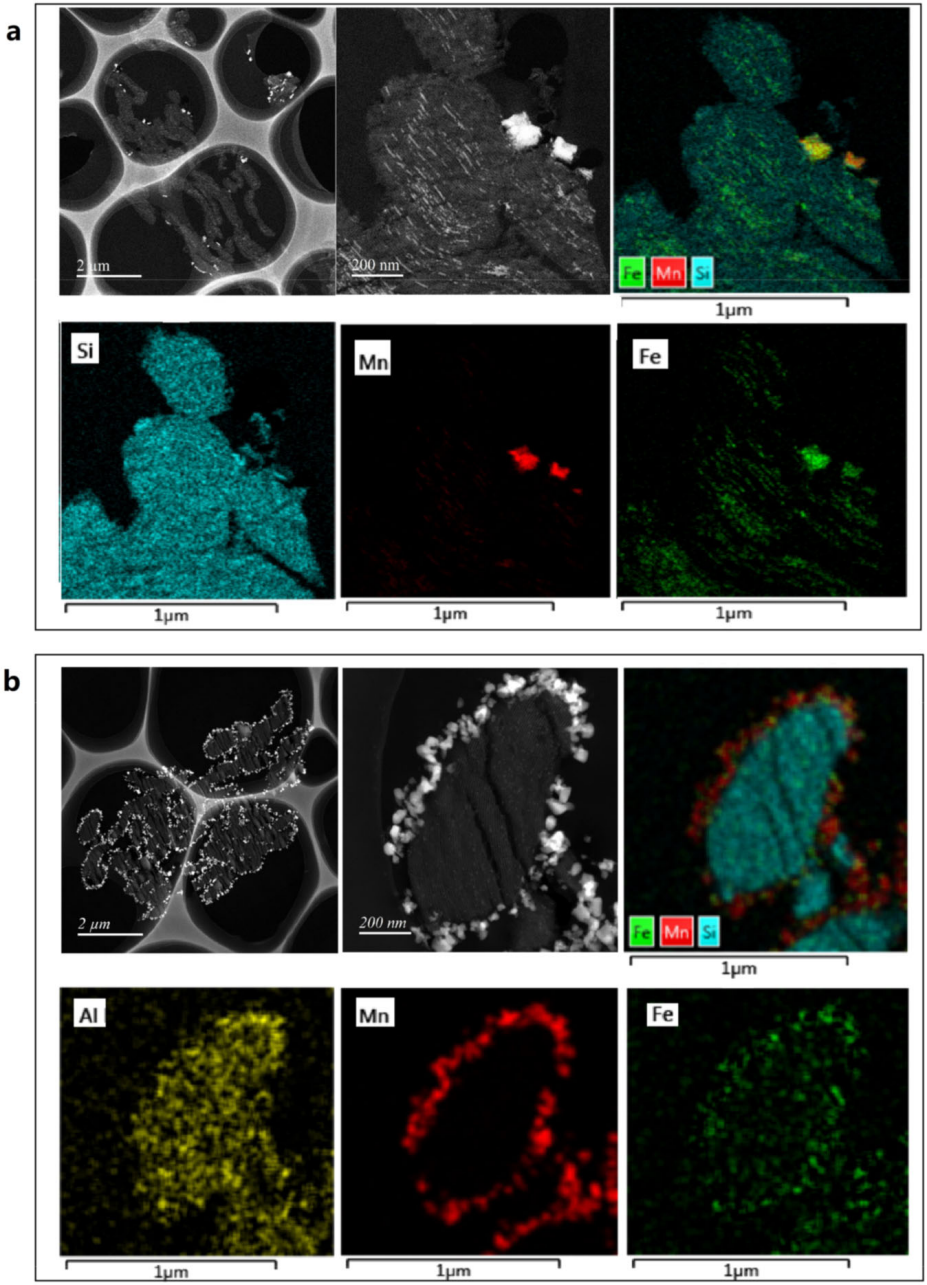
HAADF-STEM images of the molecular sieve catalysts. **a** Fe–Mn/SBA-15 and **b** Fe–Mn/Al-SBA-15.

Nanometric Fe and Mn species can be identified according to their different contrasts in the HAADF-STEM images. Figure 5 evidences that the active components can highly dispersed both in Fe–Mn/SBA-15 catalyst and Fe–Mn/Al-SBA-15 catalyst. In the Fe–Mn/SBA-15 catalyst, both Fe and Mn species were present in the pores of the molecular sieve with few outside the pores. The oxide particles in the pores were ~2–5 nm in size. As a comparison, the oxide particles outside the pores were much bigger of 60–80 nm in size. As shown in Fig. 5b, Al species were uniformly dispersed on the molecular sieve framework and extra-framework in the Fe–Mn/Al-SBA-15 catalyst. Fe and Mn species also present in the pores of the molecular sieve and outside the pores. However, the number of manganese oxide particles outside the pores is significantly increased with a greatly reduced size of 20–40 nm. This change may arise from the EFAL, which induced the crystal growth of manganese oxide outside the pores.

The redox properties of catalysts can greatly affect the NH_3_-SCR performance^35–43^. H_2_-temperature-programmed reduction (TPR) studies were therefore performed on the Fe–Mn/Al-SBA-15 and Fe–Mn/SBA-15 catalysts. The results are shown in Supplementary Fig. 4, Supplementary Table 3, and Supplementary Note 3. The results show that the area of the low-temperature reduction peak for the Fe–Mn/Al-SBA-15 catalyst was relatively large and peaks shift to low temperature. This indicates that Al doping can improve the low-temperature reducibility of the catalyst to benefit the denitrification.

**Fig. 6:**
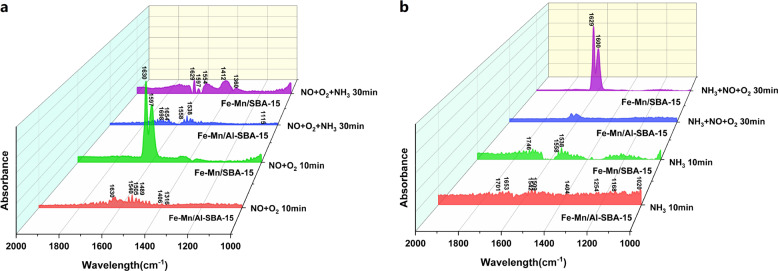
DRIFT spectra of Fe–Mn/ SBA-15 and Fe–Mn/ Al-SBA-15 catalysts. **a** Exposed to NO and O_2_ and then treated with NH_3_ at 200 °C. **b** Exposed to NH_3_ and then treated with NO + O_2_ at 200 °C.

The in situ IR spectra of the Fe–Mn/SBA-15^21^ and Fe–Mn/Al-SBA-15 catalyst were measured to understand the origin of their different catalytic performance. Figure 6a shows that three nitrate peaks at 1630 cm^−1^ (bridged bidentate nitrates), 1540 cm^−1^ (monodentate nitrates), and 1505 cm^−1^ (monodentate nitrates) appeared when 800 ppm NO and 3% O_2_/N_2_ were introduced to Fe–Mn/Al-SBA-15 sample for 10 min at 200 °C. Bidentate nitrates are formed from less stable nitrates and nitrites. The linear nitrite peak at 1489 cm^−1^, monodentate nitrite peaks at 1406 cm^−1^ and bidentate nitrite peaks at 1316 cm^−1^ also appeared^41^. Only two strong peaks appeared at 1630 and 1597 cm^−1^ were observed in DRIFT of Fe–Mn/SBA-15 sample, which are related to bridged bidentate nitrate and chelate bidentate nitrate, respectively^21^. Those nitrite peaks were not observed in the Fe–Mn/SBA-15 spectrum. This indicated that both the acidity of the catalyst and nitrite adsorption on the catalyst surface increase after Al doping. After further exposure to NO and O_2_, the intensities of these nitrite peaks gradually decreased. Specific details are available in Supplementary Fig. 5. This shows that nitrite was less stable on the surface of the catalyst, more active and easier to desorb or react compared with nitrate. According to literature reports, nitrite and nitrate are preferably decomposed to N_2_ and N_2_O, respectively^38,39^. This is one of the reasons why the N_2_ selectivity of Fe–Mn/Al-SBA-15 is better than that of Fe–Mn/SBA-15. When 800 ppm NH_3_ was then passed into the in situ reaction cell, the peaks of the nitrite all disappeared. This shows that the NH_3_ gas reacts with the nitrite on the catalyst surface. The desorption rate of nitrite increased over NH_3_ introduction time (Supplementary Fig. 5b).

Figure 6b shows in situ IR spectra of the Fe–Mn/Al-SBA-15 sample under a flow of 800-ppm NH_3_ at 200 °C. After a 10 min gas flow, peaks from NH_3_ at Lewis acidic sites also appeared at 1168, and 1254 cm^−1^, and NH_4_^+^ peaks at Brønsted acidic sites appeared at 1404, 1653, and 1701 cm^−1^. The vibration peak at 1020 cm^−1^ corresponds to weakly adsorbed or gas phase NH_3_ peaks. The peaks from surface-adsorbed −NH_2_ also appeared at 1509 and 1542 cm^−1^. With increasing exposure time, the peak intensities of the NH_3_ species became increasingly weak, which indicated that these species were not stable on the catalyst surface. The fast desorption of NH_3_ species will favor SCR reaction (Supplementary Fig. 5c). In the Fe–Mn/SBA-15 sample, fewer peaks are observed at 1538 cm^−1^ (surface-adsorbed −NH_2_), 1558 cm^−1^ (NH_3_ at Lewis acidic sites) and 1746 cm^−1^ (NH_4_^+^ peaks at Brønsted acidic sites). This supports the NH_3_-TPD result that the acid center of Fe–Mn/SBA-15 catalyst is less than that of Fe–Mn/Al-SBA-15 catalyst (Fig. 4e). When 800 ppm NO and 3% O_2_ were then passed into the in situ reaction cell, the peaks of nitrate at 1600 and 1629 cm^−1^ appeared in Fe–Mn/Al-SBA-15 sample, respectively. The peak intensities of these two peaks are higher in the Fe–Mn/SBA-15 catalyst. This also shows that the nitrate formed on the Fe–Mn/ SBA-15 catalyst is more stable than that on the Fe–Mn/Al-SBA-15 catalyst. The absence of nitrite peaks suggests that nitrite could be quickly reacting with adsorbed NH_3_ species and desorption immediately after it is formed on the surface of the catalyst^43^.

All DRIFT spectra of transient (Supplementary Fig. 5e) and steady-state reactions (Supplementary Fig. 5f) on Fe–Mn/Al-SBA-15 catalyst demonstrate that NH_3_ at Lewis acidic sites and NH_4_^+^ at Brønsted acidic sites had high catalytic activities, and −NH_2_ and –NH_4_NO_2_ were the intermediate species in the reaction. The transient reaction of NO + O_2_ resulted in the adsorption of nitrite and weakly adsorbed species in the gas phase as NO active species. The SCR reaction, therefore, mainly occurred through the two adsorbed states of NH_3_ and nitrite, and weakly adsorbed NO in the gas phase. The Langmuir–Hinshelwood and Eley–Rideal mechanisms may be simultaneously involved. All DRIFT spectra of Fe–Mn/SBA-15 catalyst are shown in Supplementary Fig. 6. The possible SCR reaction routes on Fe–Mn/Al-SBA-15 catalyst are illustrated in Supplementary Fig. 7.

Under practical application conditions, the exhaust gases contain small amounts of SO_2_ and H_2_O that can poison catalyst. This is because SO_2_ can be catalytically oxidized to SO_3_ by the catalyst, then SO_3_ can react with catalysts to irreversibly form metal sulfates or ammonium sulfates, that is, NH_4_HSO_4_ and (NH_4_)_2_SO_4_. These ammonium sulfates can physically block the catalyst and most of the active sites, which results in the declined NO_x_ conversion. Therefore, the influence of SO_2_ and H_2_O on the catalytic performance was investigated. The results shown in Supplementary Fig. 8 suggest that the NO_x_ conversion decreased to 64.79% in 1 h in the presence of 500 ppm of SO_2_ and 5% H_2_O. The catalyst slightly recovered after stopping the feed of H_2_O and SO_2_. However, the NO_x_ conversion only maintained at 65–70%. Although the denitrification efficiency decreases intermediately after the treatment of H_2_O and SO_2_, it remains stable over the time. In industrial applications, the reduced airspeed may increase the denitrification efficiency.

The low-temperature NH_3_-SCR performance of reported deNO_x_ catalysts is summarized in Supplementary Table 4. Supplementary Table 4 and Supplementary Note 4 show similar denitrification efficiency compared with the reported low-temperature denitrification catalysts. However, we can achieve the same performance using faster gas hourly space velocity (GHSV) and lower gas concentrations in comparison with previous studies. Moreover, the developed catalysts of this work are considerably less costly and free of V. They are, thus, more suitable for industrial applications.

### Density functional theory (DFT) calculations

To understand the role of Al in the catalytic process, DFT calculations were carried out^44–50^. The XRD data revealed that the introduction of Al caused the Mn-based species to change from MnO_2_ to Mn_2_O_3_. To validate the role of Al, the phase change energies were calculated based on the following reactions:

2 $$2{\mathrm{Mn}}_2{\mathrm{O}}_3 + {\mathrm{O}}_2 = 4{\mathrm{MnO}}_2,$$

3 $${\mathrm{2Al}}_{0.125}{\mathrm{Mn}}_{1.875}{\mathrm{O}}_3 + {\mathrm{O}}_2 = 4{\mathrm{Al}}_{0.0625}{\mathrm{Mn}}_{0.9375}{\mathrm{O}}_2,$$

4 $$2{\mathrm{Si}}_{0.125}{\mathrm{Mn}}_{1.875}{\mathrm{O}}_3 + {\mathrm{O}}_2 = 4{\mathrm{Si}}_{0.0625}{\mathrm{Mn}}_{0.9375}{\mathrm{O}}_2.$$

The reaction energies for these three reactions were 0.040, 0.174, and −0.046 eV, respectively. Here, the negative values suggest that the reaction is thermodynamically allowed. The reaction energy of the first reaction suggested that Mn_2_O_3_ is slightly more stable than MnO_2_. Even after the doping of a low concentration of Al of 6.25%, the reaction energy was more than four times larger than that without any dopant, which indicates that the Al can further stabilize the Mn_2_O_3_ polymorph. However, the MnO_2_ became more stable when 6.25% Si dopants are introduced. The thermodynamic analyses explain why the MnO_2_ became the dominant species when a SBA-15 support only with Si cations is employed and the Mn_2_O_3_ phase is the main Mn-based species after Al is introduced in the support as evidenced by the XRD image (Fig. 4d).

**Fig. 7:**
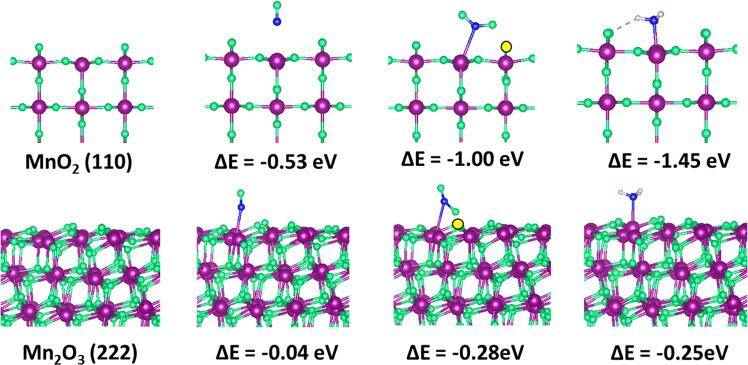
The adsorption of NO and NH_3_ on MnO_2_ (110) and Mn_2_O_3_ (222) surfaces. Color code: purple: Mn, green: O, yellow: O vacancy, blue: N, and gray: H.

The N_2_ selectivity of NH_3_-SCR catalyst was directly related to the interaction strength of the reactants and intermediates with the surface. DFT calculations on the adsorption properties of relevant small molecules on MnO_2_ (110) and Mn_2_O_3_ (222) surfaces were performed. These two surfaces were selected because they may be the most exposed facets from the XRD result. Only the Mn species were focused for this comparative study because previous experimental results suggest that they play the decisive role for the N_2_ selectivity in the SCR reaction^21^. The adsorption properties of NO and NH_3_ were calculated, since both are the reactants. The configuration of adsorbed NO_2_ with an adjacent O vacancy was also considered because it was one of the important intermediates during the SCR reaction. Indeed, the total energies of the configuration of NO_2_ with an O vacancy on both surfaces are lower than that with adsorbed NO on the clean surfaces, which suggested that the adsorbed NO could easily be oxidized by the crystal oxygen. After the formation of NO_2_, the adsorption energy of NO_2_ on the defective surface with the oxygen vacancy is close to zero, which indicates that the NO_2_ on the surface is considerably mobile to form N_2_O_4_ for further reduction, as illustrated in Fig. 7.

## Discussion

Fly ash-derived Al-SBA-15 MMS with different Si:Al molar ratios were prepared by a post-synthesis impregnation method without water. The effects of pH value and the amount of Al added on the structure of the molecular sieve were investigated. The results show that Al is successfully doped in SBA-15 MMS. The acidity (acid position, acidic strength, L acid/B acid ratio, and L acid and B acid synergy) were investigated by in situ FTIR with the transmission mode, Py-IR, TMP-^31^P MAS NMR, ^13^C acetone-^1^H/^13^C CP MAS, ^1^H/^27^Al TRAPDOR NMR, and two-dimensional ^1^H-^1^H DQ-MAS NMR. All characterization results confirm that Al exists not only in the framework but also in the extra-framework, which correspondingly provided B acid and L acid to the catalyst. The strength of L acid was greater than the strength of B acid. The spatial proximity of L acid and B acid enhanced their synergistic effect and greatly enhanced their acidity.

The denitrification activities of a series of SCR catalysts were investigated. At 150–300 °C, the denitrification efficiency of the Fe–Mn/Al-SBA-15 catalyst was higher than 90%. Moreover, the N_2_ selectivity of the Fe–Mn/Al-SBA-15 catalyst remained above 86% at 150–300 °C, which was higher than that of the Fe–Mn/SBA-15 catalyst. This was the result of a combination of acidity, redox properties, and synergistic effects in the molecular sieve catalysts. The XRD, XPS, HAADF-STEM, and DFT results show that Al dopants can induce the growth of Mn_2_O_3_ catalysts, which is beneficial for NH_3_-SCR. The denitrification reaction mechanism for the Fe–Mn/Al-SBA-15 catalyst at 200 °C was investigated by in situ IR spectroscopy. The results showed that many nitrite peaks were present after doping with Al, which further indicated that Al doping increased the catalyst acidity and improved nitrite adsorption on the catalyst surface. The E–R and L–H mechanisms were simultaneously involved, and the E–R mechanism dominated.

Based on the DFT results, there is a stronger interaction between NO and MnO_2_, which indicates that Fe–Mn/SBA-15 possesses a strong reactivity for NO conversion. This matches the reported experimental data^21^. However, the adsorption strength of NH_3_ on the MnO_2_ (110) surface is too strong, which limits its reduction capability. As a comparison, the adsorption energy of NH_3_ on Mn_2_O_3_ (222) was −0.25 eV, which benefits the desorption of NH_3_ to selective produce N_2_. The DFT data also confirm the DRIFT analysis conclusion that NH_3_ is unstable on the Fe–Mn/Al-SBA-15 catalyst with respect to that on the Fe–Mn/SBA-15 catalyst. The desorption of NH_3_ is a crucial step in the SCR reaction for the selectively produce N_2_. This explains the high N_2_ selectivity when Mn_2_O_3_ acts as the active site in a catalyst. Thus, the Al-induced component engineering for the formation of Mn_2_O_3_ is essential for the advance of low-temperature NH_3_-SCR.

## Methods

### Synthesis of fly ash-derived SBA-15 MMS and AlCl_3_ powders

The chemical composition and powder XRD pattern of high-alumina fly ash are shown in Supplementary Method (Supplementary Fig. 9 and Supplementary Note 5). Hydrochloric acid was used to extract alumina in the fly ash. The Al leaching rates from fly ash were investigated for various grinding times and acid concentrations, as shown in Supplementary Fig. 10, along with a detailed explanation in Supplementary Notes 6 and 7. The main chemical components of the extracted Al solution were Al_2_O_3_ 357.41 g/L, Fe_2_O_3_ 7.69 g/L, CaO 10.75 g/L, MgO 0.95 g/L, and SiO_2_ 0.178 g/L. The filter cake obtained by extracting Al from the fly ash was Si rich. Its main chemical components were 80.51% SiO_2_, 13.34% Al_2_O_3_, 0.25% CaO, and 0.32% Fe_2_O_3_. The main phases were mullite, quartz, and anatase. The chemical composition of the Si-rich filter cake was similar to that of the fly ash; therefore, we used the experimental conditions previously used for Si extraction by a fly ash alkali dissolution method for Si-leaching experiments. Details are reported in our previous publications^19,20^.

The SBA-15 MMS synthesized hydrothermally at 100 °C had a three-dimensional porous structure (Supplementary Figs. 11 and 12, Supplementary Notes 8 and 9). Main pore channels were produced and disordered microporous wall and mesoporous tunnels were obtained. The BET surface area of the prepared fly ash-derived SBA-15 MMS was 793.59 m^2^/g, the pore volume was 0.748 cm^3^/g, and the average pore diameter was 6.11 nm. The main chemical components of the SBA-15 MMS were SiO_2_ 98.81%, Na_2_O 0.53%, Fe_2_O_3_ 0.006%, P_2_O_5_ 0.003%, Cl 0.40%, K_2_O 0.004%, SO_3_ 0.22%, and others 0.03%.

The main chemical components of the resulting AlCl_3_ crystals were Al_2_O_3_ 97.41%, Fe_2_O_3_ 0.69%, CaO 0.005%, MgO 0.0015%, and Cl 22.6%. Supplementary Fig. 13 and Supplementary Note 10 show that the prepared AlCl_3_ crystal was mainly composed of AlCl_3_·6H_2_O, and no other impurity peaks appeared, which indicated that the prepared AlCl_3_ crystals were relatively pure.

### Synthesis of fly ash-derived Al-SBA-15 MMS

A series of Al-SBA-15 catalysts were prepared through a impregnation method with prepared AlCl_3_ crystals as the Al precursors and the resulting fly ash-derived SBA-15 MMS as the support; various molar ratios of Si:Al were used. A certain quality of AlCl_3_ crystals and solvent (H_2_O/absolute ethanol) were mixed with SBA-15 (1 g) and the mixture was stirred for 24 h at 60 °C. After, the mixture was dried at 100 °C for 10 h, and then calcined in air at 550 °C for 5 h. The heating rate was 5 °C/min. Al-SBA-15 MMS with various Si:Al molar ratios were obtained. It should be noted that the Si/Al ratios were calculated from chemical stoichiometric compositions used in the sample syntheses.

### Synthesis of Fe–Mn/Al-SBA-15 MMS catalyst

An Fe–Mn/Al-SBA-15 MMS catalyst was prepared by the same method. Al-SBA-15 (1 g) was added to Mn(NO_3_)_2_ and Fe(NO_3_)_2_ solutions at a Mn:Fe molar ratio of 1:1. The resulting materials were denoted by *m*Fe-*n*Mn/Al-SBA-15, where *m* and *n* represent the Fe and Mn weight percentage loadings, respectively.

**Fig. 8:**
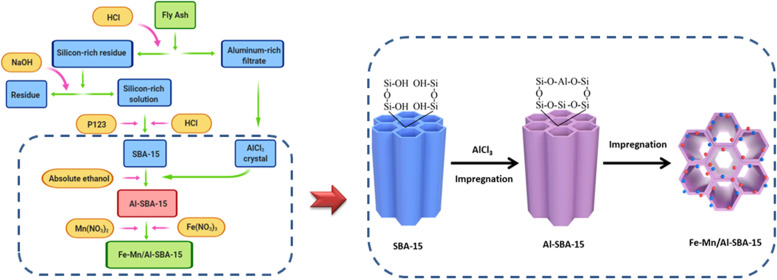
Preparation of fly ash-derived Fe–Mn/Al-SBA-15. Schematic illustration for preparation process of Fe–Mn/Al-SBA-15 catalyst from fly ash. The dotted box on the right is a schematic diagram of the dotted box on the left.

The fly ash-derived Fe–Mn/Al-SBA-15 preparation route is shown in Fig. 8. Fe–Mn/SBA-15 materials were also prepared for comparison.

### Characterizations

XRD was performed with a D8 ADVANCE diffractometer (Bruker, Germany), using Cu Kα radiation and a step size of 0.02°. Small-angle data were collected from 0.5° to 5° at a scanning rate of 0.5°/min. Wide-angle data were collected from 5° to 90° at a scanning rate of 3°/min.

N_2_ adsorption–desorption isotherms were recorded using an ASAP2020 automatic physical adsorption instrument (Micromeritics, USA) at 77 K after degassing the samples at 373 K.

TEM analyses were performed on a JEOL 200F TEM/STEM microscope operating at 200 kV. The HAADF images and elemental mapping were taken on a JEM-200F TEM (JEOL Co. Ltd) equipped with a Cs probe corrector (CEOS, Gmbh) and a 100 mm^2^ active area EDS detector (X-Max^N^100TLE, Oxford Instruments plc). Before observation, the SBA-15 powders were embedded in Epoxy resin, cut to 50 nm thin slices by ultramicrotome (EM UC7, Leica Microsystems Inc), and transferred onto holey carbon film.

XPS analysis was performed on an ESCALAB 250xi (Thermo Scientific, UK) using monochromatic Al Kα radiation (1486.6 eV) operating at 25 W.

H_2_-TPR experiments were performed with an AutoChem II 2920 instrument (USA). The catalyst (0.3 g) was pretreated in an Ar flow (50 mL/min) for 30 min at 500 °C to remove water and other impurities. As the samples cooled, the Ar flow was replaced by a reductive mixture of 10.0% H_2_ in Ar and the reactor temperature was raised to 800 °C at a heating rate of 10 °C/min.

NH_3_-TPD experiments were performed with an automatic physical and chemical adsorption instrument (AutoChem II 2920, Micromeritics). Before adsorption of NH_3_ at 373 K, the samples were heated at 773 K in a He flow. The amount of NH_3_ desorbed between 373 and 873 K at a heating rate of 10 K/min was determined with an on-line gas chromatograph equipped with a thermal conductivity detector.

^27^Al and ^29^Si MAS NMR spectra were recorded with a Bruker Avance III HD/89 mm instrument. Tetraethyl orthosilicate and Al(H_2_O)_6_^3+^ were used as the references for ^29^Si and ^27^Al, respectively.

Prior to solid-state NMR experiments, the Al-SBA-15 samples were placed in glass tubes and dehydrated at 673 K under a pressure below 2.0 Pa for 10 h on a vacuum line. After the dehydrated samples were cooled to room temperature, 2.4 molecule/u.c. of 2-^13^C-acetone was introduced and frozen by liquid N_2_. Saturated adsorption of TMP onto the Al-SBA-15 samples was carried out in a similar way. Finally, the samples upon adsorption of the probe molecule as well as the dehydrated Al-SBA-15 samples were flame sealed. The sealed samples were transferred into a ZrO_2_ rotor (tightly sealed by a Kel-F cap) under a dry nitrogen atmosphere in a glove box.

Solid-state NMR experiments were carried out on a Bruker Avance III 500 spectrometer with a 4-mm triple-resonance MAS probe. The Larmor frequencies were 500.6, 125.9, and 202.6 MHz for ^1^H, ^13^C and ^31^P nuclei, respectively. ^1^H spin echo MAS NMR spectra were acquired with a *π*/2 pulse length of 5.0 μs and a recycle delay of 2 s. The ^1^H/^27^Al TRAPDOR experiment was carried out with a spinning speed of 14 kHz, an irradiation time of 143 μs, and a radio frequency field strength of 50 kHz for ^27^Al. In the 2D ^1^H-^1^H DQ-SQ MAS NMR experiments, double-quantum coherences of 286 μs and a spinning speed of 14 kHz were applied. The increment interval in the indirect dimension was set to 71.4 μs. 128 t1 increments and 96 scan accumulations for each t1 increment were used. ^13^C CP/MAS NMR experiments were performed with a contact time of 1.2 ms, a recycle delay of 2 s, number of scans of 6000, and MAS of 14 kHz. ^31^P CP/MAS NMR experiments were performed with a contact time of 1.5 ms, a recycle delay of 2 s, number of scans of 1000, and MAS of 10 kHz. The chemical shifts of ^1^H and ^13^C were externally referenced to adamantine, whereas the ^31^P chemical shifts were calibrated using (NH_4_)_2_HPO_4_.

In situ transmission IR sample activation conditions: the sample was a self-supporting piece with a diameter of 13 mm. The temperature was increased at a rate of 10 °C/min from room temperature to 500 °C, held for 60 min, rapidly reduced to 350 °C, the kept stable for 5 min sampling (resolution 4 cm^−1^, number of scans 64).

DRIFT spectra were recorded with an IR Prestige-21 instrument (Shimadzu) at a resolution of 4 cm^−1^ and averaged over 500 scans. These studies were performed by heating pre-calcined powder samples in situ from room temperature to 673 K at a heating rate of 5 K/min in a pure N_2_ flow (40 mL/min). The samples were kept at 673 K for 3 h and then cooled to 323 K. Py vapor (20 μL) was then introduced under a N_2_ flow; IR spectra were recorded at various stages of Py desorption, which was maintained by evacuation at progressively higher temperatures (323–473 K). A resolution of 4 cm^−1^ was attained after averaging 500 scans for all the IR spectra recorded. XPS was performed with an ESCALAB 250xi instrument (Thermo Scientific, UK), using monochromatic Al Kα radiation (1486.6 eV), at 25 W. The sample was outgassed overnight at room temperature in an ultrahigh-vacuum chamber (<5 × 10^−^^7^ Pa). All binding energies were referenced to the C 1 s peak at 284.6 eV. The experimental errors were within ±0.1 eV.

### SCR activity measurements

The SCR reaction was evaluated in a fixed-bed reactor. The sample (0.3 g) was put in a reaction tube and the tube was placed in simulated flue gas for 2 h. The gas mixture contained 300 ppm NO, 300 ppm NH_3_, and 3% O_2_, with N_2_ as the balancing gas; the GHSV was ~120,000 h^−1^. The catalytic activity was determined by analyzing the inlet and outlet gases with a flue gas analyzer (MultiGas™ 6030, MKS) at temperatures between 100 and 300 °C. The NO_x_ conversion and N_2_ selectivity were calculated as follows:

5 $${\mathrm{NO}}_{\rm{x}}\,{\mathrm{conversion}} = \left[ {\left( {[{\mathrm{NO}}_{\rm{x}}]_{{\mathrm{in}}} - [{\mathrm{NO}}_{\rm{x}}]_{{\mathrm{out}}}} \right)/[{\mathrm{NO}}_{\rm{x}}]_{{\mathrm{in}}}} \right] \times 100\%,$$

6 $${\mathrm{N}}_2\,{\mathrm{selectivity}} = \left( {1 - \frac{{2\left[ {{\mathrm{N}}_2{\mathrm{O}}} \right]_{{\mathrm{out}}}}}{{\left[ {{\mathrm{NO}}_{\rm{x}}} \right]_{{\mathrm{in}}} + \left[ {{\mathrm{NH}}3} \right]_{{\mathrm{in}}} - \left[ {{\mathrm{NO}}_{\rm{x}}} \right]_{{\mathrm{out}}} - \left[ {{\mathrm{NH}}3} \right]_{{\mathrm{out}}}}}} \right) \times 100\%,$$

where NO_x_ is the sum of the NO and NO_2_ concentrations, [NO_x_]_out_ is the outlet concentration of NO_x_, [NO_x_]_in_ is the inlet concentration of NO_x_, [NH_3_]_out_ is the outlet concentration of NH_3_, [NH_3_]_in_ is the inlet concentration of NH_3_, and [N_2_O]_out_ is the outlet concentration of N_2_O.

### DFT calculations

The spin-polarization DFT computations were conducted using the Vienna ab initio simulation package based on the projector augmented wave (PAW) method^48–50^. Electron–ion interactions were described using standard PAW potentials, with valence configurations of 3p^6^4s^2^3d^5^ for Mn, 2s^2^2p^4^ for O (O), 2s^2^2p^3^ for N, and 1s^1^ for H. A plane-wave basis set was employed to expand the smooth part of wave functions with a cut-off kinetic energy of 520 eV. The exchange and correlation functional parameterized by Perdew–Burke–Ernzerhhof^48–50^, a form of the general gradient approximation, was used throughout. Owing to the large Coulombic repulsion between localized d electrons of transition metals, the DFT+U method was used to correct the material properties of transition metal oxides, particularly for magnetic ground states and electronic structures. For Mn, the U–J value was set as 3.5 eV.

The MnO_2_(110) and Mn_2_O_3_ (222) were simulated using the 12-layer slab model with the consideration of their magnetic structures. A sufficiently large vacuum region of 15 Å was used to ensure that the periodic images were well separated. The bottom six layers were fixed at the bulk position. The other atoms were allowed to relax during the structural optimization. The (2 × 3) and (1 × 1) surface cells were employed for MnO_2_(110) and Mn_2_O_3_ (222), respectively, for the study on the adsorption of NO and NH_3_ with one adsorbate molecule on the topmost surface layer. The corresponding k-point grids were gamma-centered (4 × 4 × 1) and (2 × 2 × 1) for MnO_2_(110) and Mn_2_O_3_ (222), respectively. The properties of NO, NO_2_, and NH_3_ molecules were calculated in a 20 × 20 × 20 Å^3^ box. The convergence criterion for the electronic self-consistent loop was set to 10^−5^ eV. The atomic structures were optimized until the residual forces were below 0.002 eV Å^−1^. The adsorption energy can be defined as follows:

7 $$\Delta E_{{\mathrm{ads}}} = E_{{\mathrm{gas}}/{\mathrm{substrate}}} - E_{{\mathrm{substrate}}} - E_{{\mathrm{gas}}},$$

where *E*_gas/substrate_ is the total electronic energy of the substrate with an adsorbed gas molecule and *E*_substrate_ and *E*_gas_ correspond to the energy of the substrate and the gas molecule, respectively, in a vacuum.

## Supplementary information

Peer Review File

Supplementary Information

## Data Availability

The data supporting the findings of this study are available within the article and its Supplementary Information files, or from the corresponding authors on reasonable request.
