# Supplementary material for: Aluminium-induced component engineering of mesoporous composite materials for low-temperature NH3-SCR
Source: Commun Chem. 2020 May 27;3:66. doi: 10.1038/s42004-020-0311-4 (PMC9814655; doi:10.1038/s42004-020-0311-4)
Supplement: Supplementary file 2 — Supplementary Information [file 42004_2020_311_MOESM2_ESM.pdf]

Supplementary Information

Aluminium-Induced Component Engineering of Mesoporous Composite Materials for Low-Temperature NH_3-SCR

Ge Li[1†], Baodong Wang[1,* †], Ziran Ma[1], Hongyan Wang[1], Jing Ma[1], Chunlin Zhao[1], Jiali Zhou[1], Dehai Lin[1], Faquan He[1], Zhihua Han[1], Qi Sun[1,2*] & Yun Wang[3*]

[1] *National Institute of Clean-and-Low-Carbon Energy, Beijing 102211, China*

[2] *Institute for Sustainable Energy and Resources, Qingdao University, Shandong, 266071, China*

[3] *Centre for Clean Environment and Energy, Gold Coast Campus, Griffith University, Queensland 4222, Australia*

*Corresponding authors. *E-mail addresses*: baodong.wang.d@chnenergy.com.cn (B. D. Wang); qisun_l@hotmail.com.com (Q. Sun); yun.wang@griffith.edu.au (Y. Wang).

† These authors contributed equally to this work.

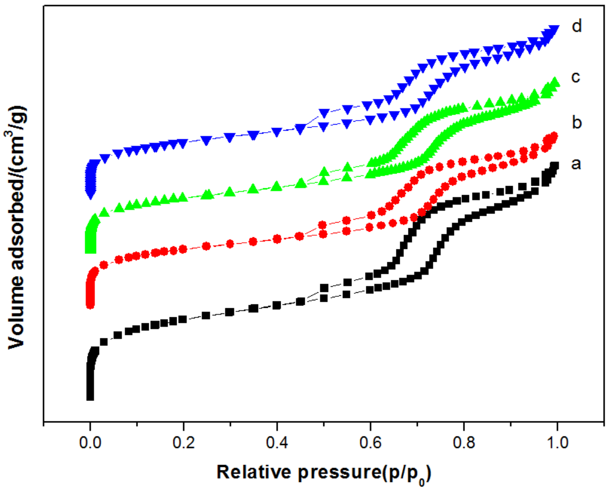

Supplementary Fig. 1 N_2 adsorption–desorption isotherms of (a) SBA-15, (b) Al-SBA-15 (Si/Al = 8.35), (c) Al-SBA-15 (Si/Al = 4.17), and (d) Al-SBA-15(Si/Al = 2.09).

Supplementary Notes 1. Supplementary Fig. 1 shows that the N_2 adsorption–desorption isotherms of all the SBA-15 molecular sieves, whether pure silica or Al-doped molecular sieves, were typical type IV isotherms with H1-type hysteresis loops. There were two clear inflection points in the relative pressure range of 0.6–0.90, with sharp capillary condensation at the middle of the inflection point. The two inflection points corresponded to the mesopore distribution range and the sharpness of the capillary condensation indicated whether the mesopore distribution was concentrated. Supplementary Fig. 1 shows that the mesopore distribution of the SBA-15 molecular sieves was narrow. Grafting different amounts of Al did not change the hysteresis loop shape and mesopore distribution. The structural properties of all the molecular sieve catalysts are shown in Supplementary Table 1. The data showed an average pore diameter of 6.14 nm and a BET specific surface area of 795.39 m^2/g for the SBA-15 molecular sieves. The pore volume, pore size, and BET specific surface area decreased with an increasing amount of grafted Al. This was because during grafting, Al entered the pore walls of the sieves and occupied part of the pore space. In addition, some of the Al may have been present as extra-framework Al, which led to a decrease in the pore volume, pore size, and BET specific surface area. These results are consistent with those reported in the literature [1-4].

Supplementary Table 1 BET surface areas and pore size distributions for Al-SBA-15

Sample	Pore size (nm)	BET surface area (m^2/g)	Pore volume (cm^3/g)
SBA-15	6.14	795.39	0.748
Al-SBA-15(Si/Al =8.35)	5.16	680.25	0.603

Al-SBA-15(Si/Al =4.17)	5.02	575.34	0.566
Al-SBA-15(Si/Al =2.09)	4.99	558.62	0.447

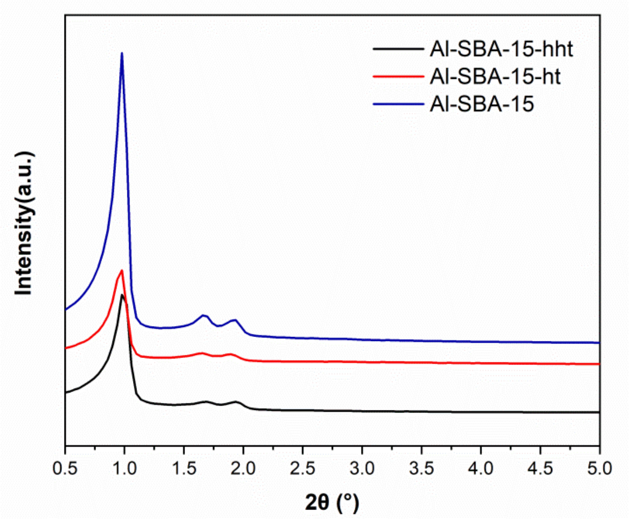

Supplementary Fig. 2 XRD patterns of Al-SBA-15 samples after different treatments

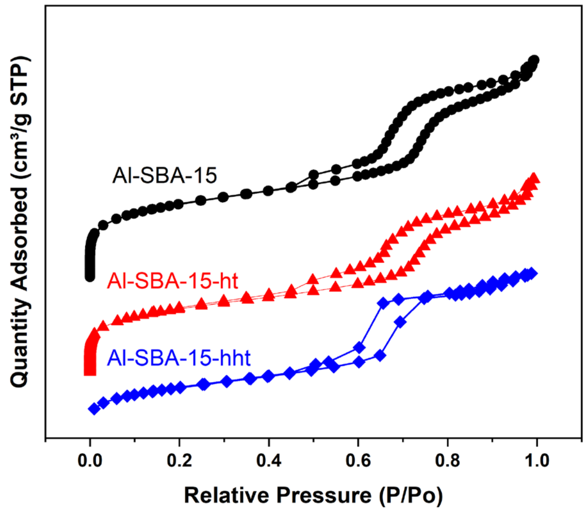

Supplementary Fig. 3 N_2 adsorption-desorption isotherms of Al-SBA-15 samples after different treatments

Supplementary Table 2 BET surface areas and pore size distributions for Al-SBA-15 samples after different treatments

Sample	Pore size (nm)	BET surface area (m^2/g)	Pore volume (cm^3/g)
Al-SBA-15	5.02	575.34	0.566
Al-SBA-15-ht	4.91	563.82	0.543
Al-SBA-15-hht	4.13	418.67	0.489

Supplementary Notes 2. Since hydrothermal stability is the biggest factor limiting the application of molecular sieves, the determination of their hydrothermal stability is necessary. Previous studies usually use the structure and specific surface area to determine the stability

of molecular sieves after processing in boiling water at 100 °C. However, their hydrothermal stability needs to be examined at 600-800 °C if molecular sieves are used in the petrochemical industry. In our study, we performed the hydrothermal stability test by treating the obtained Al-SBA-15 molecular sieve in a closed bottle at 100 °C for 300 h under static conditions. The samples after hydrothermal aging are termed as Al-SBA-15-ht. High-temperature hydrothermal stability was tested by treating the sample at 600 °C for 6 h in a flow of 100% water vapor, which are termed as Al-SBA-15-hht. The XRD spectra of Al-SBA-15 after hydrothermal treatment at 100 °C for 300 h and 600 °C for 6 h are shown in Supplementary Fig. 2. The N_2 adsorption-desorption isotherms are shown in Supplementary Fig. 3, and the structural properties are shown in Supplementary Table 2. From Supplementary Fig. 2, three distinct diffraction peaks (100), (110), and (200) were still observed in the XRD spectrum after the heat treatment at 100 °C and 600 °C, which indicated that treated Al-SBA-15 remained in its original hexagonal structure. However, after hydrothermal aging, the peak intensities of Al-SBA-15-ht and Al-SBA-15-hht decreased slightly with the increased peak width. Moreover, the peak of the Al-SBA-15-hht molecular sieve after high-temperature hydrothermal aging was obviously shifted to the higher angle, which indicated that the pore size of the mesopore had been reduced. From the N_2 adsorption-desorption isotherm and Supplementary Table 2, it can be seen that the shape of the hysteresis ring was similar to that of Al-SBA-15 after hydrothermal treatment at 100 °C. However, the amount of N_2 adsorption, the specific surface area, the pore volume and pore diameter slightly decreased. In addition, the peak shape of the Al-SBA-15-hht molecular sieve was obviously different from that of Al-SBA-15. After high-temperature hydrothermal aging at 600 °C, the adsorption amount of N_2 significantly decreased. The shape of the micropores was also changed. The specific surface area, pore volume, and pore diameter significantly decreased, which indicates that the porous structure of the Al-SBA-15 molecular sieve was destroyed after the high temperature hydrothermal treatment. The higher skeletal silicon-aluminum ratio is responsible for the decrease in its high temperature hydrothermal stability. This is one of the reasons why silicalite MMS cannot be used in the petroleum catalytic cracking industry. Our results are consistent with previous researches[1-3].

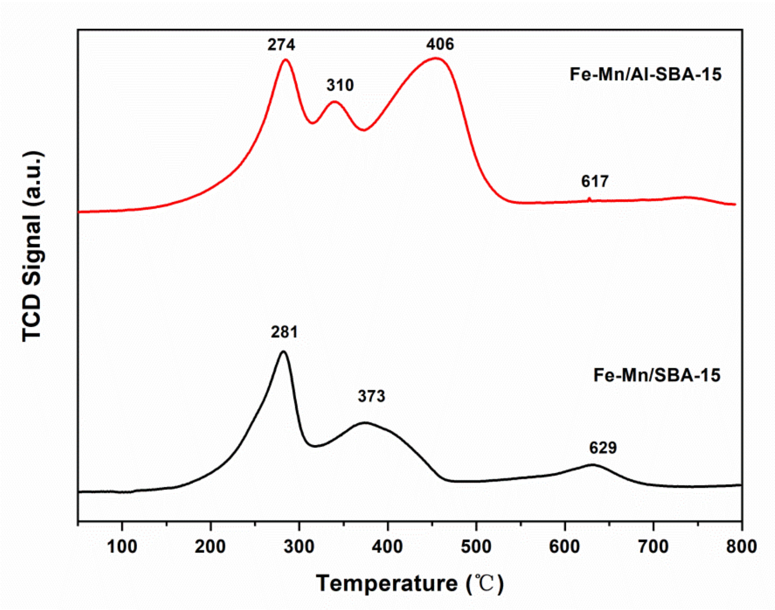

Supplementary Fig. 4 H_2-TPR curves for Fe-Mn/Al-SBA-15 and Fe-Mn/SBA-15 catalysts.

Supplementary Notes 3. The redox properties of metal oxide-supported catalysts greatly affect the NH_3-SCR performance[5-8]. H_2-TPR studies were therefore performed on the Fe-Mn/Al-SBA-15 and Fe-Mn/SBA-15 catalysts. The results are shown in Supplementary Fig. 4. The H_2-TPR curves for the Fe-Mn/Al-SBA-15 and Fe-Mn/SBA-15 catalysts exhibited several reduction peaks in the low-temperature range, i.e. 200–500 °C. There were several MnO_x peaks at 274, 281, and 310 °C, and these were attributed to the gradual reduction of MnO_x ($MnO_2 \rightarrow Mn_2O_3 \rightarrow Mn_3O_4$). The peak at 373 °C was attributed to the gradual reduction of Fe_xO_y, i.e. $Fe_2O_3 \rightarrow FeO$. The low-temperature peak for Fe-Mn/Al-SBA-15 shifted to a lower temperature compared with that for Fe-Mn/SBA-15. This indicated that doping with Al increased the low-temperature reducibility of the catalyst and increased the denitrification activity. This also explained why the denitrification activity of Fe-Mn/Al-SBA-15 was better than that of the Fe-Mn/SBA-15 catalyst. The denitrification temperature window for Fe-Mn/Al-SBA-15 was extended to a lower temperature, which indicated that the introduction of an appropriate amount of Al species could increase the reducibility of the catalyst. The reduction peak at 406 °C for Fe-Mn/Al-SBA-15 was presumed to be a superposition of the reduction peaks for $Mn_3O_4 \rightarrow MnO$ and Fe_2O_3. The reduction peaks at 617 and 629 °C may correspond to FeO reduction ($FeO \rightarrow Fe^0$). It has been reported in the literature that a low-temperature reduction peak is related to a better redox performance by the catalyst[5-8]. The reduction peak was therefore fitted and the area was calculated. The results showed that the area of the low-temperature reduction peak for the Fe-Mn/Al-SBA-15 catalyst

was relatively large. This value may be high because doping with Al increased the amount of active components on the molecular sieve surface. This is reflected in the XPS results. The presence of more active components on the catalyst surface increased the opportunity for redox reactions with H_2; therefore, the redox performance of the Fe-Mn/Al-SBA-15 catalyst was enhanced. This is also consistent with the relatively high NH_3-SCR low-temperature denitrification activity observed for the Fe-Mn/Al-SBA-15 catalyst.

Supplementary Table 3 Reduction peak temperatures and H_2 consumptions for Fe-Mn/Al-SBA-15 and Fe-Mn/SBA-15 catalysts

Samples	Temperature peak(℃)				H_2 consumption(mmol/g)				
	Peak 1	Peak 2	Peak 3	Peak 4	Peak 1	Peak 2	Peak 3	Peak 4	Total
Fe-Mn/Al-SBA-15	274	310	406	617	0.16	0.25	0.67	0.07	1.15
Fe-Mn/ SBA-15	281	373	629	—	0.09	0.18	0.40	—	0.67

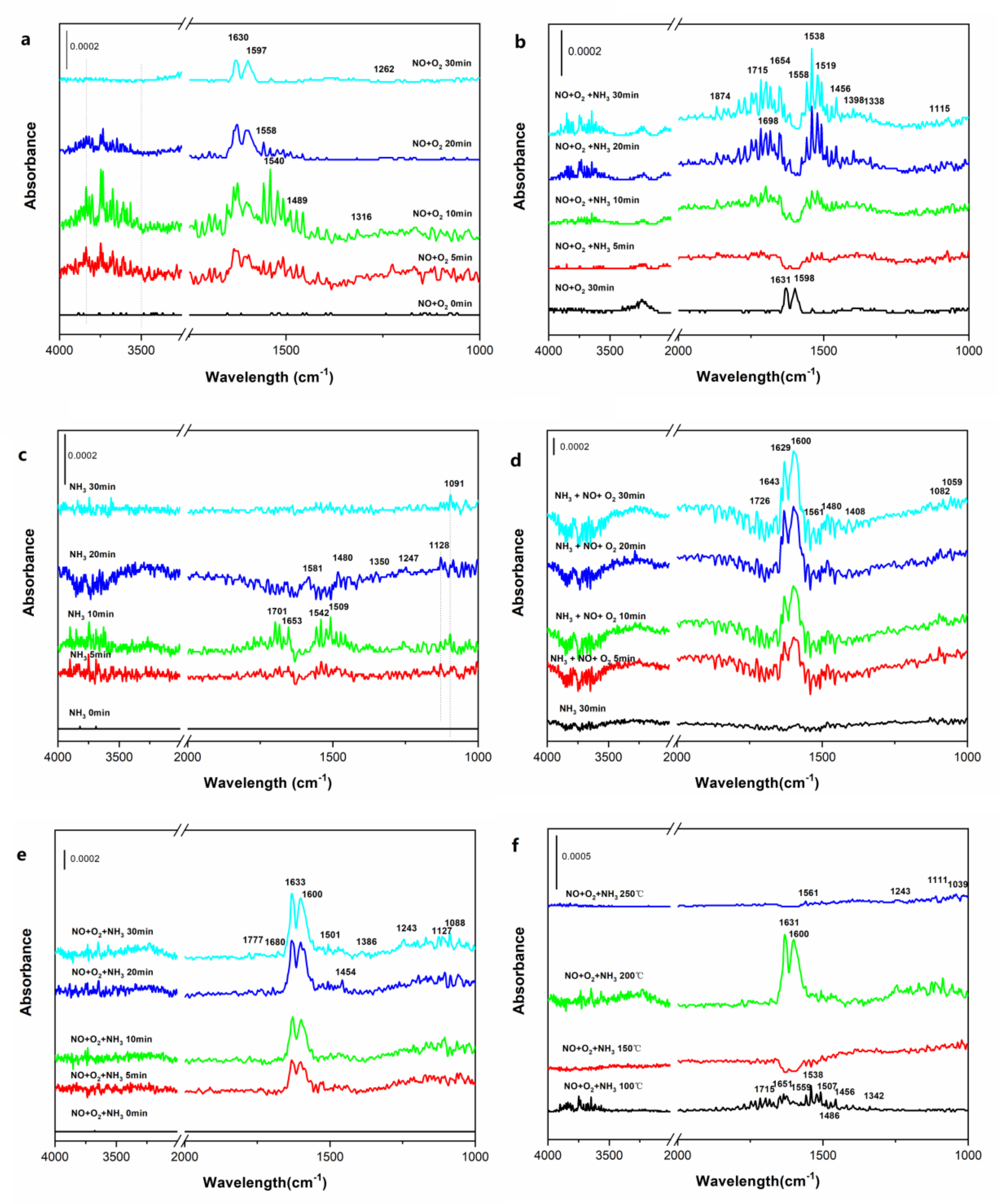

Supplementary Fig. 5 DRIFT spectra of Fe-Mn/ Al-SBA-15 catalyst. **a** Exposed to NO and O_2 for various times. **b** Preabsorbed with NO + O_2 and then treated with NH_3. **c** Exposed to NH_3 for various times. **d** Preabsorbed with NH_3 and then treated with NO + O_2. **e** Exposed to NH_3 + NO + O_2 for 30 min. **f** With a temperature gradient in a NH_3 + NO + O_2 atmosphere.

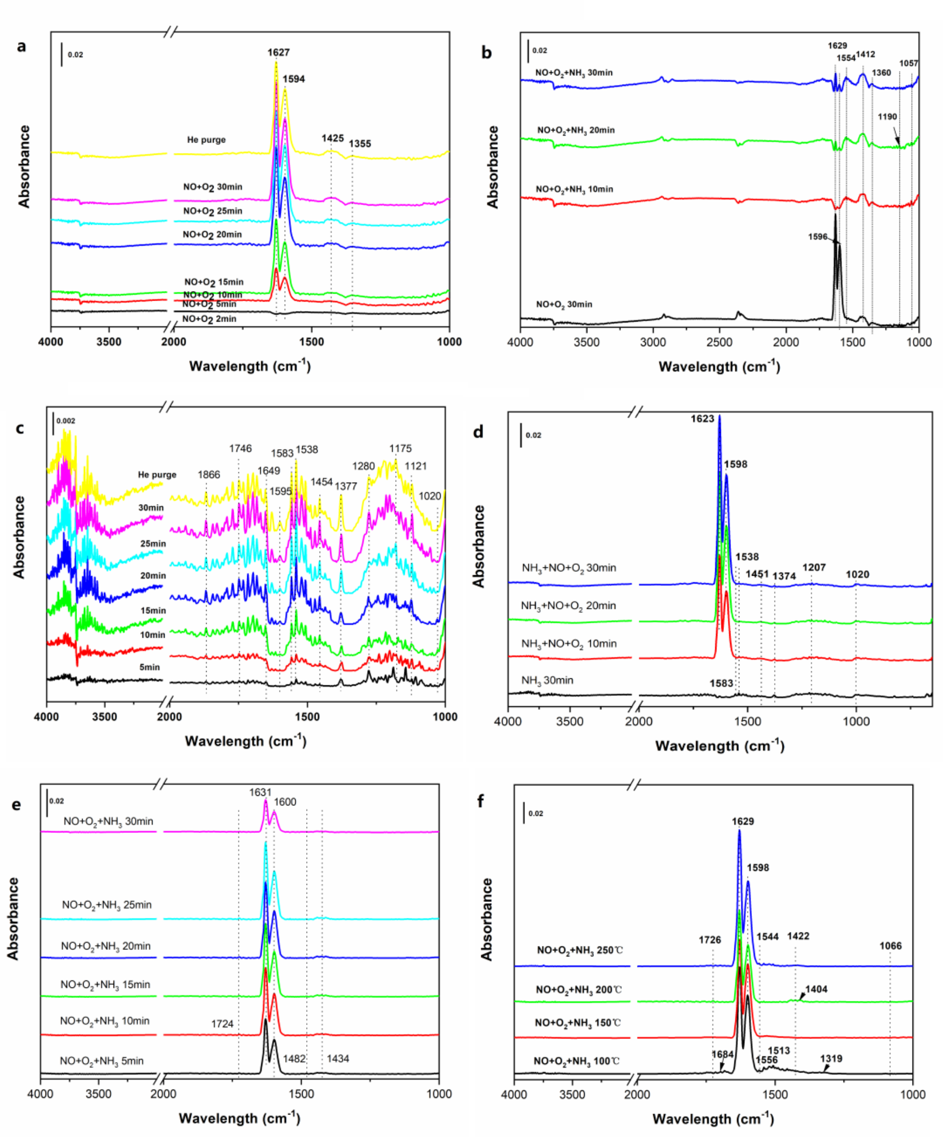

Supplementary Fig. 6 DRIFT spectra of Fe-Mn/SBA-15 catalyst. **a** Exposed to NO and O_2 for various times. **b** Preabsorbed with NO + O_2 and then treated with NH_3. **c** Exposed to NH_3 for various times. **d** Preabsorbed with NH_3 and then treated with NO + O_2. **e** Exposed to NH_3 + NO + O_2 for 30 min. **f** With a temperature gradient in NH_3 + NO + O_2 atmosphere[21].

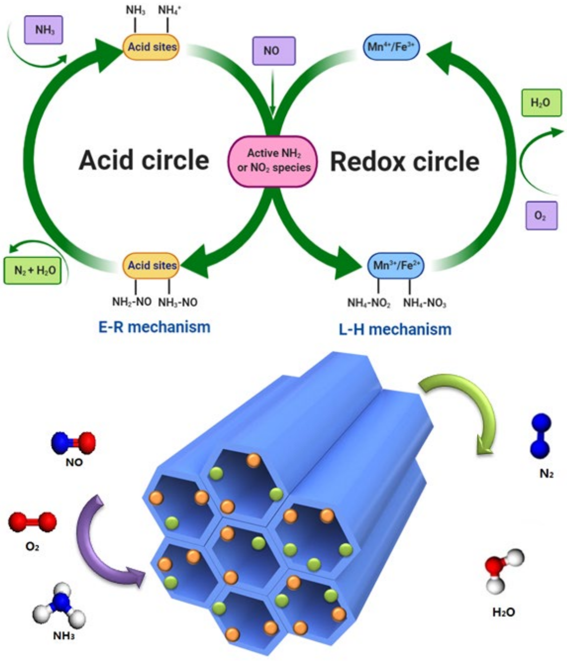

Supplementary Fig. 7 Possible reaction routes of NH_3-SCR reaction gas on the surface of catalysts.

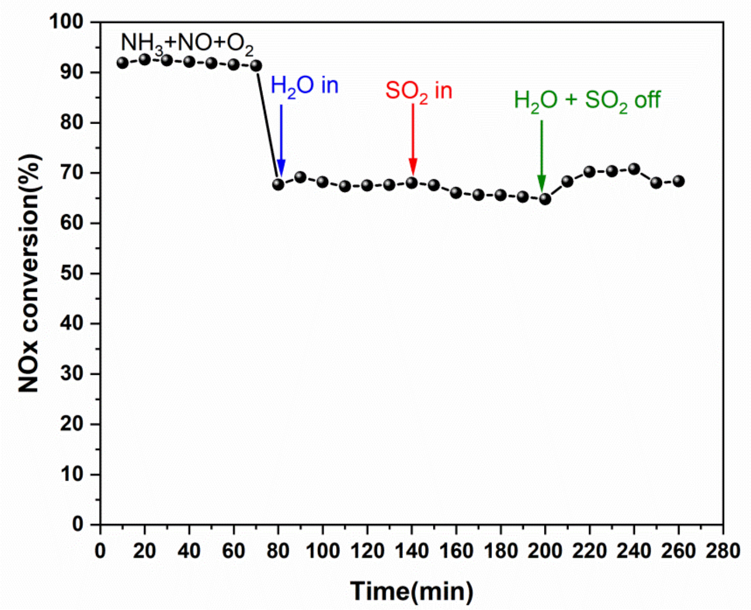

Supplementary Fig. 8 Effect of water and/or SO_2 on/off presence in the feed stream and the long-term stability of the Fe-Mn/Al-SBA-15 catalyst at 200 °C. Reaction conditions: [NO]=[NH_3]=300 ppm; [SO_2]= 500ppm; [H_2O]=5 vol%; [O_2]=3 vol%.

**Supplementary Table 4** Comparison of low-T NH_3-SCR efficiency with different deNOx catalysts.				
Catalyst	Feed Composition	GHSV	NO Conversion/%	Ref.
15Fe-Mn/ZSM-5	600 ppm NO, 600 ppm NH_3, 5.0 % O_2, 100 ppm SO_2	45,000 h^{-1}	90% (100-250℃)	9
12.5Ce-FeMnO_x	0.1% NO, 0.1% NH_3, 3% O_2	30,000 h^{-1}	95% (100-140℃)	10
FeMnTiO_x	800 ppm NO, 800 ppm NH_3, 5.0 % O_2	30,000 h^{-1}	100% (100-350℃)	11
$Fe_{0.3}Ho_{0.1}Mn_{0.4}/TiO_2$	0.08 % NO, 0.08 % NH_3, 5% O_2	20,000 h^{-1}	90% (120-200℃)	12
K- α-MnO_2	500 ppm NO, 500 ppm NH_3, 5%O_2, 10% H_2O,100 ppm SO_2	60,000h^{-1}	100% (150-250℃)	13
MnO_x-CeO_2 nanosphere	500 ppm NO, 500 ppm NH_3, 5% O_2	60,000 h^{-1}	100% (125-250℃)	14
MnO_x-TiO_2 NS	1000 ppm NO, 1100 ppm NH_3, 4% O_2	50,000 h^{-1}	80% (200-300℃)	15
Cu-Fe-Ti<br>Co-Fe-Ti	500 ppm NO, 500 ppm NH_3, 3.5 % O_2	60,000 h^{-1}	90% (200-250℃)	16
Fe-Mn/SBA-15	300 ppmNO, 300 ppm NH_3,3%O_2	120,000h^{-1}	90% (200-250℃)	17
Fe-Mn/Al-SBA-15	300 ppmNO, 300 ppm NH_3,3%O_2	120,000h^{-1}	90% (150-300℃)	This study

Supplementary Notes 4. Our Fe-Mn/Al-SBA-15 shows similar denitrification efficiency compared with the reported low-temperature denitrification catalysts. However, we can achieve the same performance using faster gas hourly space velocity (GHSV) and lower gas concentrations in comparison with previous studies. According to the E-R and L-H mechanisms, the higher initial gas concentration in the flue gas can lead to the more gaseous reactants actively adsorbed on the surface of the catalyst, which can promote the removal of NO and further increase the denitrification efficiency. In previous studies, the GHSV was relatively low of 20,000h^{-1} to 60,000 h^{-1} since the lower GHSV can improve the denitrification efficiency. This is because of the increase of GHSV means the rate of gas molecules passing through the interface increases. As a result, the residence time of gas molecules on active surface or active center of per unit volume in catalyst is shortened. This causes the smaller average adsorption rate of reactants, which reduces the denitrification efficiency. However, we can achieve the same catalytic performance with faster GHSV (120,000 h^{-1}) and lower gas concentrations here. It demonstrates that our obtained Fe-Mn/Al-SBA-15 catalyst has excellent low-temperature catalytic denitrification performance. Moreover, it is conceivable that in our experiment, if the GHSV is reduced, the denitrification efficiency will be close to 100%.

Supplementary Methods

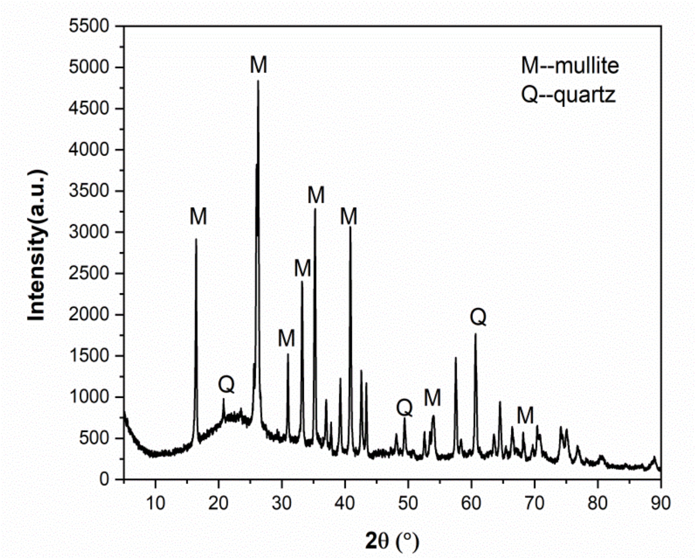

Supplementary Fig. 9 Powder XRD pattern of high-alumina fly ash.

Supplementary Notes 5. High-alumina fly ash from a power plant in Inner Mongolia (China) was used; its chemical composition (mass percentage) was as follows: 53.17% SiO_2, 36.86% Al_2O_3, 2.58% Fe_2O_3, 2.48% TiO_2, and 2.27% CaO. The powder XRD pattern of the fly ash (Supplementary Fig. 9) shows “bun”-shaped peaks, which were mainly associated with aluminosilicate glass phases, and crystalline phases, mainly mullite and quartz.The principle of material balance and the chemical composition of the high-alumina fly ash (FA-01) were used to calculate the phase composition of the ash using the LINPRO program. The fly ash contained mullite 15.9%, quartz 3.7%, glass phases 67.1%, rutile 0.9%, and hematite 4.7%. Hydrochloric acid (analytical grade, 36-38%), sodium hydroxide (analytical grade, ⩾96%), ferric nitrate (analytical grade, $Fe(NO_3)_3 \cdot 9H_2O$) and manganese nitrate (analytical grade) were all supplied by the Sinopharm Chemical Reagent Co., Ltd. (Beijing, China), ethanol absolute (analytical grade, ⩾ 99.7%) was provided by the Beijing Enoch Technology Co., Ltd. (China), and polyethylene glycol–polypropylene glycol–polyethylene glycol (P123) was from Aldrich. Hydrochloric acid was used to extract the alumina in fly ash because the silica in fly ash does not react with this acid. This enabled effective separation of alumina and silica in the fly ash and prevented Si from entering the Al solution. However, alumina in fly ash does not exist independently. It is present in glass and mullite together with silica; therefore, acid hydrolysis of the fly ash involved a reaction

of the acid with mullite and glass. The alumina in the glass is amorphous; therefore, its leaching was thermodynamically possible. However, literature reports and patents show that the extraction rate of alumina by hydrochloric acid decomposition of fly ash is not high; the rate of the reaction between alumina and the acid is low at room temperature; therefore, no reaction with acid occurs under macroscopic conditions. Acid leaching can be promoted by adding a cosolvent or changing the heating method. However, these methods are not suitable for industrial production. In terms of dynamics, the reaction of an acid-soluble mineral is a liquid–solid reaction. The rate of a liquid–solid reaction is mainly controlled by diffusion and the chemical reaction rate. The diffusion rate can be increased by stirring and raising the temperature. The chemical reaction rate can be increased by increasing the temperature. A reduction in the size of the solid-phase particles increases the solid–liquid contact area and can also increase the reaction rate. Fly ash activation by grinding was therefore used to increase the reaction rate in the high-temperature acid leaching of fly ash. Concentrated hydrochloric acid was selected as the leaching agent, and the Al leaching rate from fly ash was investigated for various grinding times and acid concentrations.

Supplementary Notes 6. Supplementary Fig. 10a shows the Al leaching rate when the molar ratio of alumina in the fly ash to hydrochloric acid was 1:6 for different fly ash milling times. The figure shows that as the grinding time increased, the leaching rate of Al in the fly ash gradually increased. This was because the fly ash particles formed a grit after grinding to a size different from the original. The coarse lumps were crushed into fine granules, the number of broken bonds increased, the specific surface area increased, and the reaction contact surface increased. The chemical activity of the fly ash powder therefore increased. However, when the milling time exceeded 120 min, the Al leaching rate did not change much, and was approximately 87.5%. Fly ash milled for 2 h was therefore used as the raw material in subsequent experiments. The effects of the amount of hydrochloric acid on Al extraction from fly ash was investigated by performing leaching experiments with different molar ratios of hydrochloric acid to Al_2O_3. Supplementary Fig. 10b shows the Al leaching rates for various amounts of added acid. According to the stoichiometric ratio, the theoretical value for calculating the molar ratio of Al_2O_3 in fly ash to hydrochloric acid should be 6:1. Supplementary Fig. 10b shows that the Al leaching rate increased significantly with increasing the molar ratio of Al_2O_3 in the fly ash to hydrochloric acid. The leaching rate increased to more than 90% when the molar ratio was above 7:1. A higher amount of

hydrochloric acid added resulted in a higher H^+ concentration in the system; this promoted the breakage of Al–O–Si bonds and facilitated leaching of alumina in glass.

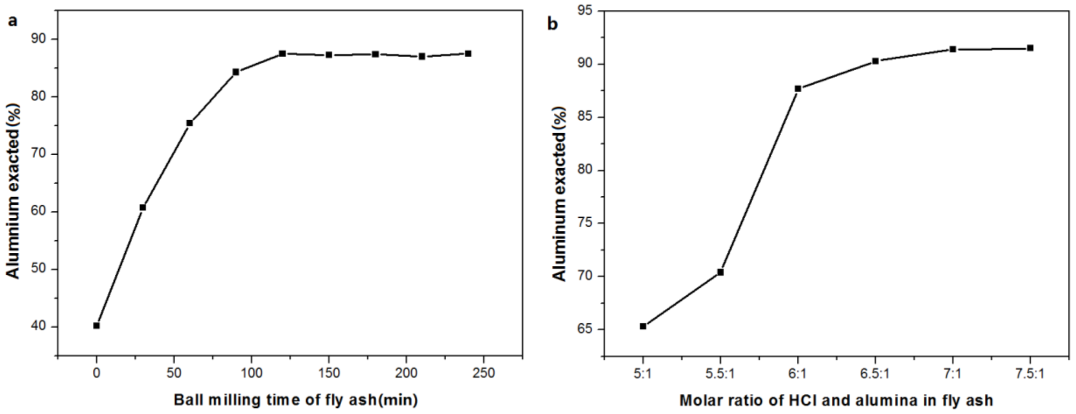

Supplementary Fig. 10 Al extraction rates from fly ash. **a** With various milling times. **b** With various molar ratios of hydrochloric acid to alumina in fly ash.

Supplementary Notes 7. Fly ash and hydrochloric acid were mixed in molar ratios of fly ash alumina to hydrochloric acid of 1:5–1:7.5 and hydrothermally reacted at 150 °C for 1–3 h. After cooling, the mixture was filtered. The filtrate was an Al salt solution with an Al_2O_3 content of 262 g/L, and the residue was Si rich. The Si-rich filter residue, sodium hydroxide, and H_2O were mixed at mass ratios of 100:60:40 and hydrothermally reacted at 95 °C for 30 min. After cooling, the mixture was filtered. The filtrate was a sodium silicate solution with an SiO_2 content of 378 g/L. Next, 2 mol/L of hydrochloric acid was added to the alkali-containing filtrate (sodium silicate solution, 30 mL) to adjust the pH to 3–5. P123 (2.35 g) was added and the mixture was reacted under mechanical stirring at 35 °C for 10 h. The mixture was then poured into a polytetrafluorethylene-lined stainless-steel reactor and crystallized at 110 °C for 48 h. The resulting mixture was filtered, washed, and dried at 95 °C for 12 h. The dried sample was calcined at 550 °C for 6 h at a heating rate of 5 °C/min. Cooling yielded powdery SBA-15 molecular sieves.

Supplementary Notes 8. The SBA-15 MMS synthesized hydrothermally at 100 °C had a three-dimensional porous structure. The main pore channels were produced with disordered microporous and mesoporous tunnels. The porous structure and structural characteristics of the fly-ash-derived SBA-15 molecular sieves are shown in Supplementary Fig. 11.Supplementary Fig. 11a shows the small-angle XRD pattern of SBA-15. The XRD pattern contained three peaks,

which were indexed to the (100), (110), and (200) reflections, and corresponded to P6mm hexagonal symmetry. Supplementary Fig. 11b shows the wide-angle XRD pattern of SBA-15. Supplementary Fig. 11c and Supplementary Fig. 11d shows the N_2 adsorption–desorption curve and pore size distribution, respectively, of the SBA-15 molecular sieves. Figure 1b shows that the molecular sieves gave a type IV curve, according to the IUPAC classification; this suggested a mesoporous structure. The N_2 adsorption–desorption curve showed an H1-type hysteresis loop in the relative pressure range p/p_0 = 0.4–0.8, which was caused by capillary condensation. The pore size distribution in Supplementary Fig. 11d indicated that the material had a highly ordered mesoporous structure, uniform pore size distribution, and regular pores. The Brunauer–Emmett–Teller (BET) surface area of the prepared fly-ash-derived SBA-15 molecular sieves was 793.59 m^2/g, the pore volume was 0.748 cm^3/g, and the average pore diameter was 6.11 nm. The main chemical components of the SBA-15 molecular sieves were SiO_2 98.81%, Na_2O 0.53 %, Fe_2O_3 0.006%, P_2O_5 0.003%, Cl 0.40%, K_2O 0.004%, SO_3 0.22%, and others 0.03 %.

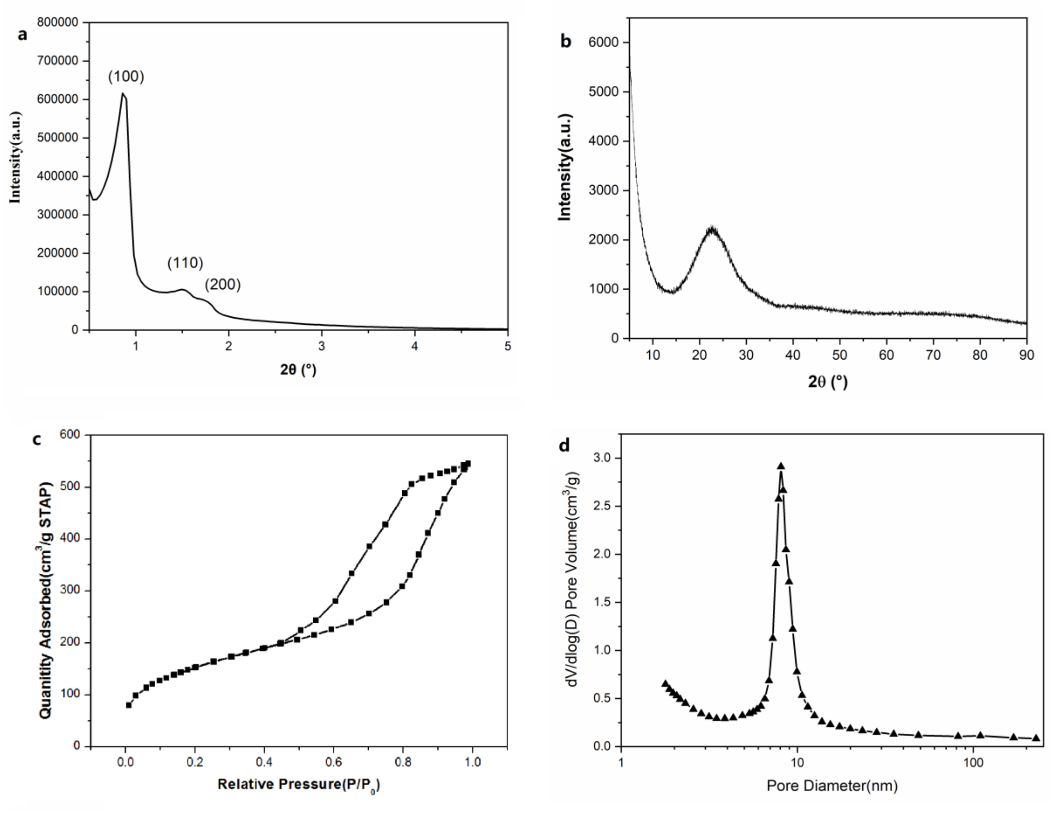

Supplementary Fig. 11 SXRD pattern, N_2 adsorption–desorption isotherms and pore size distribution of fly-ash-derived SBA-15 molecular sieves.

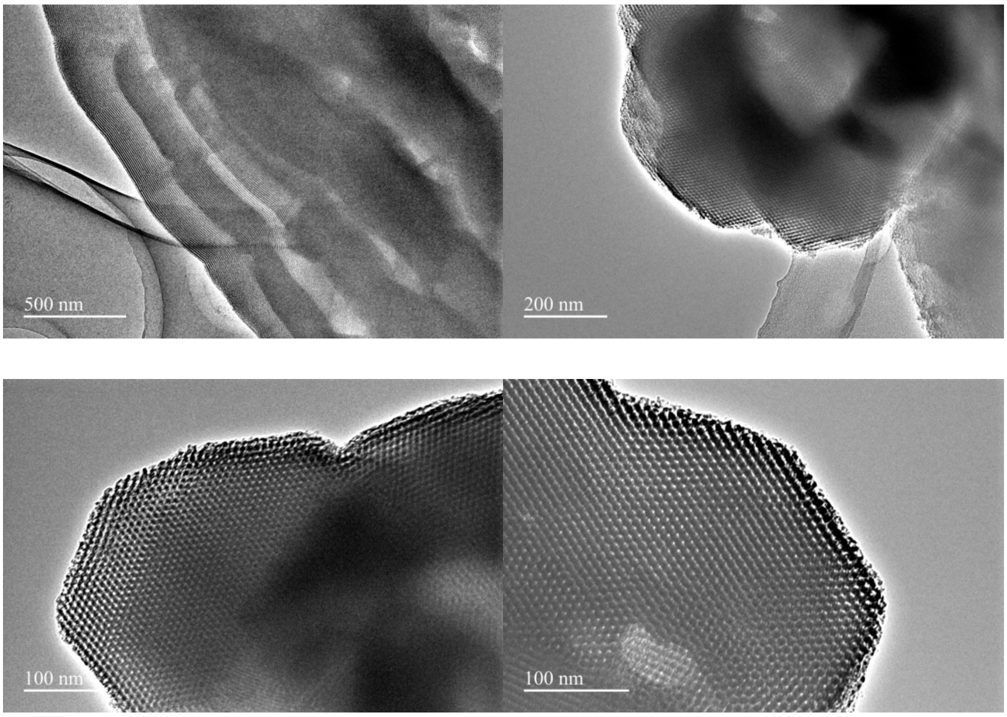

Supplementary Fig. 12 HRTEM of images of fly-ash-derived SBA-15 molecular sieves.

Supplementary Notes 9. Supplementary Fig.12 shows transmission electron microscopy images of the SBA-15 molecular sieves. The figure shows that the pores were regular and hexagonal, the skeleton had an ordered arrangement, the pore diameter was approximately 5 nm, and the thickness of the pore wall was approximately 3 nm.

Supplementary Notes 10. The obtained aluminium-containing solution was subjected to a vacuum concentration at a pressure of −0.05 MPa and a temperature of 80 °C. After concentration and cooling, the crystalline aluminium chloride was precipitated. After solid-liquid separation, a small amount of impurities, such as ferric chloride, remained in the solution owing to the low concentration. The XRD pattern of the prepared aluminium chloride is shown in Supplementary Fig. 13. The main chemical components of the $AlCl_3$ crystals were Al_2O_3 97.41 %, Fe_2O_3 0.69%, CaO 0.005%, MgO 0.0015 %, and Cl 22.6%. Figure S9 shows that the prepared $AlCl_3$ crystal was mainly composed of $AlCl_3 \cdot 6H_2O$, and no other impurity peaks appeared, which indicated that the prepared $AlCl_3$ crystals were relatively pure.

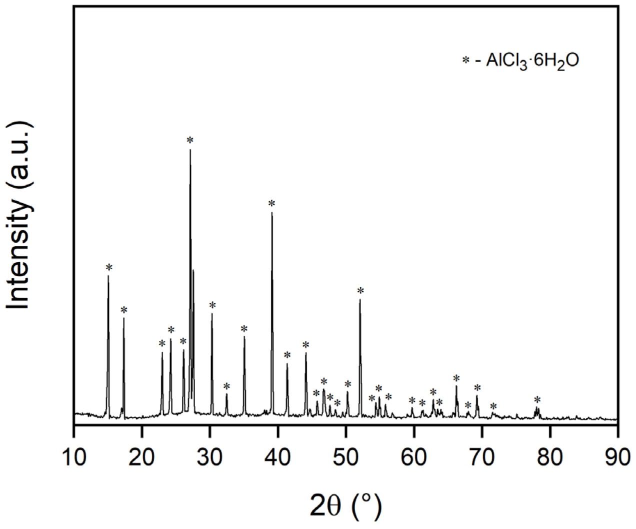

Supplementary Fig. 13 XRD pattern of fly-ash-derived $AlCl_3$ crystals.

Supplementary References

1. Impe´ror-Clerc, M., Davidson, P., Davidson, A. Existence of a microporous corona around the mesopores of silica-based SBA-15 materials templated by triblock copolymers. *J. Am. Chem. Soc.***122**, 11925-11933 (2000).

2. Ryoo, R. Birth of a class of nanomaterial. *Nature*, **575**, 40-41 (2019).

3. Nozaki, C., Lugmair, C. G., Bell, A. T., et al. Synthesis, characterization, and catalytic performance of single-site iron (III) centers on the surface of SBA-15 silica. *J. Am. Chem. Soc.* **124**, 13194-13203(2002).

4. Kockrick, E., Krawiec, P., Schnelle, W., et al. Space-confined formation of FePt nanoparticles in ordered mesoporous silica SBA-15. *Adv. Mater.* **19**, 3021-3026(2007).

5. France, L.J., Yang, Q., Li, W., et al. Ceria modified $FeMnO_x$-Enhanced performance and sulphur resistance for low-temperature SCR of NO_x. *Appl. Catal. B: Environ.* **206**, 203-215(2017).

6. Liu, J., Wei, Y.J., Li, P.Z., et al. Experimental and theoretical investigation of mesoporous MnO_2 nanosheets with oxygen vacancies for high-efficiency catalytic deNO_x. *ACS Catal.* **8**, 3865-3874(2018).

7. Qi, G., Yang, R.T. Characterization and FTIR studies of MnO_x-CeO_2 catalyst for low-temperature selective catalytic reduction of NO with NH_3. *J. Phys. Chem. B*, **108**, 5738-5747(2004).

8. Li, Y., Wan, Y., Li, Y.P., et al. Low-temperature selective catalytic reduction of NO with NH_3 over Mn_2O_3-doped Fe_2O_3 hexagonal microsheets. *ACS Appl. Mater. Interfaces*, **8**, 5224-5233(2016).

9. Mu, W.T. et al. Novel proposition on mechanism aspects over Fe-Mn/ZSM-5 catalyst for NH_3-SCR of NO_x at low temperature: rate and direction of multifunctional electron-transfer-bridge and in situ DRIFTs analysis. *Catal. Sci. Technol.*, **6**, 7532-7548(2016).

10. France, L.J. et al. Ceria modified $FeMnO_x$-Enhanced performance and sulphur resistance for low-temperature SCR of NO_x. *Appl. Catal. B: Environ.* **206**, 203-215(2017).

11. Wu, S.G., Yao, X.J., Zhang, L., et al. Improved low temperature NH_3-SCR performance of $FeMnTiO_x$ mixed oxide with CTAB-assisted synthesis. *Chem. Commun.*,**51**, 3470-3473(2015).

12. Zhu, Y.W., Zhang, Y.P., Xiao, R., et al. Novel holmium-modified Fe-Mn/TiO_2 catalysts with a broad temperature window and high sulfur dioxide tolerance for low-temperature SCR. *Catal. Commun.***88**, 64-67(2017).

13. Hao, Z.F., Shen, Z.R., Li,Y., et al. Can a poison be a gift? the role of alkali metal in the α-MnO_2 catalyzed ammonia selective catalytic reaction. *Angew. Chem. Int. Edit.* **58**, 6351-6356(2019).

14. Li, L.L., Sun, B.W., Sun, J.F., et al. Novel MnO_x-CeO_2 nanosphere catalyst for low-temperature NH_3-SCR. *Catal. Commun.* **100**, 98-102(2017).

15. Deng, S.C., Meng, T.T., Xu, B.L., et al. Advanced MnO_x/TiO_2 Catalyst with Preferentially Exposed Anatase {001} Facet for Low-Temperature SCR of NO. *ACS Catal.* **6**, 5807-5815(2016).

16. Zhu, L., Zhong, Z.P., Yang, H., et al. Comparison study of Cu-Fe-Ti and Co-Fe-Ti oxide catalysts for selective catalytic reduction of NO with NH_3 at low temperature. *J. Colloid*

Interface Sci. **478**, 11-21(2016).

17. Li, G., Wang, B.D., Sun, Q., et al. Reaction mechanism of low-temperature selective catalytic reduction of NO_x over Fe-Mn oxides supported on fly ash-derived SBA-15 molecular sieves: Structure-activity relationships and in situ DRIFTs analysis. *J. Phys. Chem. C,* **122**, 20210-20231(2018).
